# Hepatic HIF2 is a key determinant of manganese excess and polycythemia in SLC30A10 deficiency

**DOI:** 10.1172/jci.insight.169738

**Published:** 2024-04-23

**Authors:** Milankumar Prajapati, Jared Z. Zhang, Lauren Chiu, Grace S. Chong, Courtney J. Mercadante, Heather L. Kowalski, Bradley Delaney, Jessica A. Anderson, Shuling Guo, Mariam Aghajan, Thomas B. Bartnikas

**Affiliations:** 1 Department of Pathology and Laboratory Medicine, Brown University, Providence, Rhode Island, USA.; 2 Ionis Pharmaceuticals, Inc., Carlsbad, California, USA.

**Keywords:** Genetics, Hepatology, Genetic diseases, Monogenic diseases

## Abstract

Manganese is an essential yet potentially toxic metal. Initially reported in 2012, mutations in SLC30A10 are the first known inherited cause of manganese excess. SLC30A10 is an apical membrane protein that exports manganese from hepatocytes into bile and from enterocytes into the lumen of the gastrointestinal tract. SLC30A10 deficiency results in impaired gastrointestinal manganese excretion, leading to manganese excess, neurologic deficits, liver cirrhosis, polycythemia, and erythropoietin excess. Neurologic and liver disease are attributed to manganese toxicity. Polycythemia is attributed to erythropoietin excess. The goal of this study was to determine the basis of erythropoietin excess in SLC30A10 deficiency. Here, we demonstrate that transcription factors hypoxia-inducible factor 1a (Hif1a) and 2a (Hif2a), key mediators of the cellular response to hypoxia, are both upregulated in livers of Slc30a10-deficient mice. Hepatic Hif2a deficiency corrected erythropoietin expression and polycythemia and attenuated aberrant hepatic gene expression in Slc30a10-deficient mice, while hepatic Hif1a deficiency had no discernible impact. Hepatic Hif2a deficiency also attenuated manganese excess, though the underlying cause of this is not clear at this time. Overall, our results indicate that hepatic HIF2 is a key determinant of pathophysiology in SLC30A10 deficiency and expand our understanding of the contribution of HIFs to human disease.

## Abstract 2

The transcription factor HIF-2 is a key determinant of manganese excess, erythropoietin excess, and polycythemia in SLC30A10 deficiency, which underlies inherited manganese excess.

## Graphical abstract

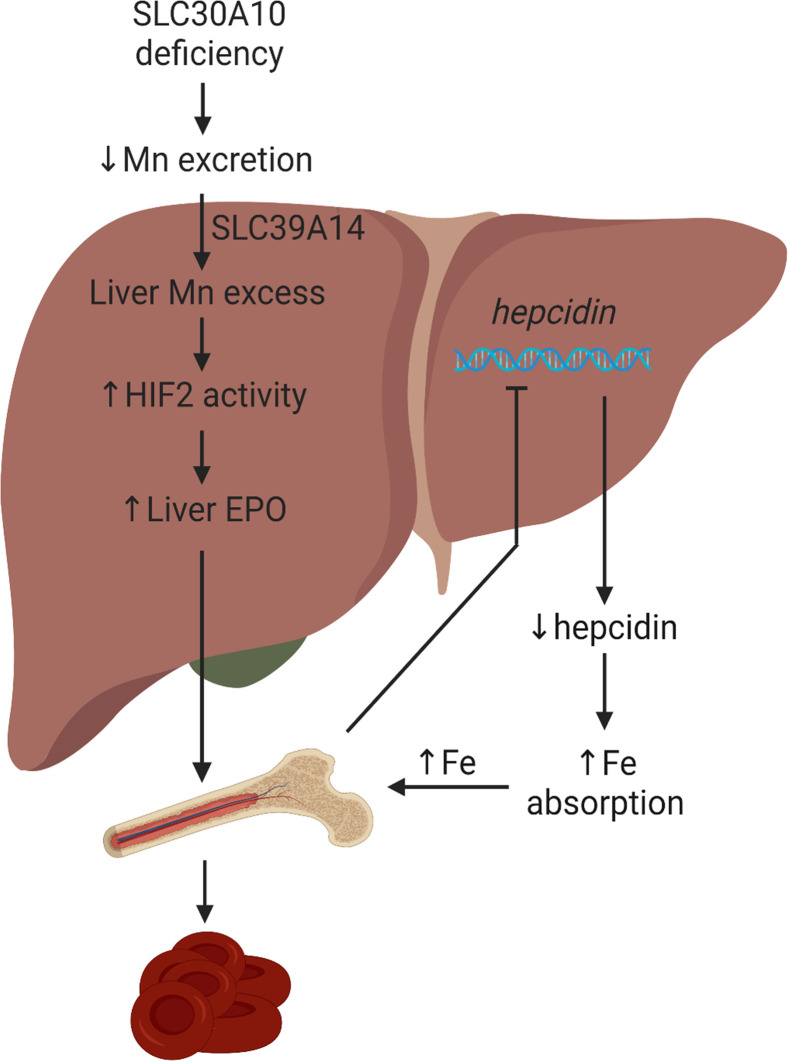

## Introduction

Manganese (Mn) is an essential yet potentially toxic metal (1, 2). It is an essential enzymatic cofactor in multiple biological processes, including antioxidant defense and protein glycosylation. When present in excess, Mn is toxic, with toxicity attributed to factors such as oxidative stress and mitochondrial dysfunction. Mn toxicity is typically caused by environmental exposure and manifests as neurologic impairment. Inherited causes of Mn excess are rare, but study of these diseases has greatly informed our understanding of the molecular basis of Mn homeostasis in the human body.

Mutations in metal transport proteins SLC30A10 (ZNT10) and SLC39A14 (ZIP14) are 2 recently described inherited causes of Mn excess. Patients with SLC30A10 deficiency were first reported in 2012, while patients with SLC39A14 deficiency were first reported in 2016 (3). SLC30A10 is an apical membrane protein that exports Mn from hepatocytes into bile and from enterocytes into the lumen of the gastrointestinal tract (4–6). SLC30A10 deficiency impairs gastrointestinal Mn excretion, resulting in severe Mn excess as well as neurologic and liver dysfunction, polycythemia, and erythropoietin (EPO) excess. Neurologic and liver disease are attributed to Mn toxicity. Polycythemia is attributed to EPO excess, but the basis of EPO excess in this disease has yet to be established. SLC39A14 is a basolateral membrane protein that imports Mn from liver sinusoids into hepatocytes and from blood into enterocytes (7–13). Like SLC30A10 deficiency, SLC39A14 deficiency impairs gastrointestinal excretion, resulting in severe Mn excess and neurologic deficits. Unlike SLC30A10 deficiency, SLC39A14 deficiency does not produce liver disease, polycythemia, or EPO excess.

As stated above, the basis of EPO excess in SLC30A10 deficiency is unknown. *EPO* is the canonical hypoxia-regulated gene. Under conditions of hypoxia, the kidneys secrete EPO to stimulate erythropoiesis in a process dependent upon transcription factors known as hypoxia-inducible factors (HIFs) (14). HIFs consist of labile α subunits and constitutively expressed β subunits. Under conditions of normoxia, HIF α subunits are subjected to prolyl hydroxylation by prolyl hydroxylases (PHDs), followed by ubiquitination and degradation. Under conditions of hypoxia, HIF α subunits are spared from degradation and can activate transcription of *EPO* and a variety of other genes. Although a role for HIFs in EPO excess and polycythemia in SLC30A10 deficiency has yet to be explored, several studies have shown links between Mn, EPO, and HIFs. Mn treatment of human airway cell line Hep2 increases HIF1α protein levels by activating MAPKs (15), though the validity of this cell line has been called into question recently (16). Mn treatment of lung cancer cells inhibits PHD activity, increases HIF1α protein levels, and stimulates expression of vascular endothelial growth factor (VEGF), a known HIF target gene (17). Mn treatment of human pulmonary epithelial cell lines increases VEGF expression, and Mn inhalation in mice increases pulmonary expression of VEGF and multiple other angiogenesis-associated genes (18). Mn treatment of human hepatoma cell line Hep3B stimulates *EPO* expression (19). A recent link between Mn, SLC30A10, and HIFs has been reported as well — Mn treatment of hepatic cell lines increases HIF1A- and HIF2A-dependent SLC30A10 expression by inhibiting prolyl hydroxylation of HIFs (20).

In this study, we investigate the link between SLC30A10 deficiency and EPO excess using Slc30a10-deficient mouse models and dietary, genetic, pharmacologic, and transcriptomic approaches. As described below, we demonstrate that hepatic Hif1a, while upregulated, has minimal impact on Epo excess and polycythemia, while hepatic Hif2a not only drives Epo excess and polycythemia but also contributes to aberrant hepatic gene expression, increased dietary iron absorption, and systemic Mn excess in Slc30a10-deficient mice. We also discuss implications of our observations for our understanding and treatment of SLC30A10 deficiency.

## Results

### Slc30a10^–/–^ mice develop Mn-dependent Epo excess and polycythemia.

**Figure 1:**
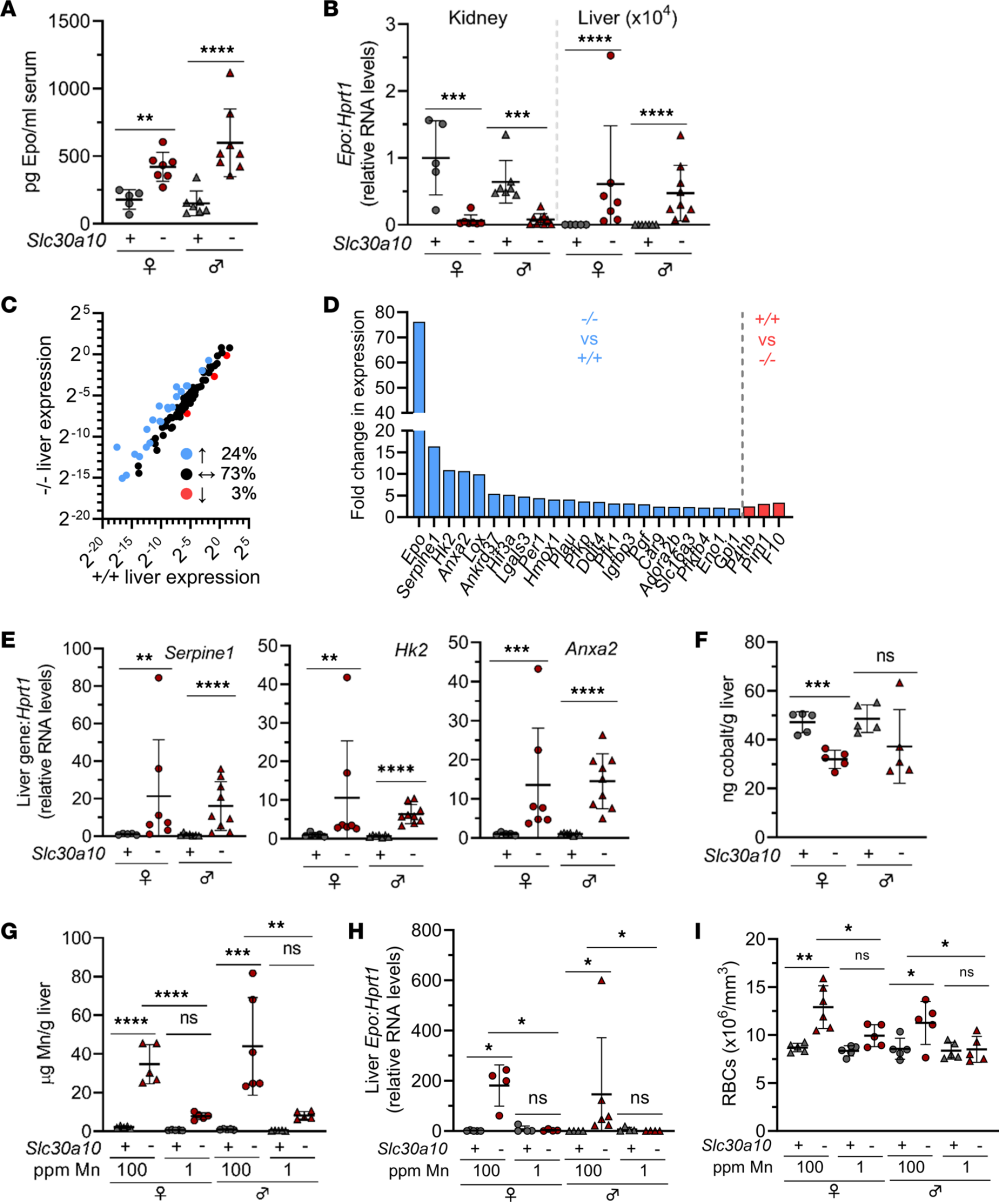
*Slc30a10^–/–^* mice develop Mn-dependent Epo excess and polycythemia. (**A** and **B**) Two-month-old *Slc30a10^+/+^* and *Slc30a10^–/–^* mice were analyzed for serum Epo levels by ELISA (**A**) and kidney and liver *Epo* RNA levels by quantitative PCR (qPCR) (**B**). (**C**–**E**) Livers from 2-month-old *Slc30a10^+/+^* and *Slc30a10^–/–^* mice were analyzed by PCR array for hypoxia-regulated genes up- (blue) or down- (red) regulated at least 2-fold (**C** and **D**), followed by qPCR validation of the top 3 differentially regulated genes besides *Epo* (**E**). (**F**) Two-month-old *Slc30a10^+/+^* and *Slc30a10^–/–^* mice were analyzed for liver cobalt levels by graphite furnace atomic absorption spectroscopy (GFAAS). (**G**–**I**) *Slc30a10^+/+^* and *Slc30a10^–/–^* mice were weaned onto Mn-sufficient (100 ppm) or -deficient (1 ppm) diets, then analyzed at 6 weeks for liver Mn levels by inductively coupled plasma optical emission spectroscopy (ICP-OES) (**G**), for liver *Epo* RNA levels by qPCR (**H**), and for RBC counts by complete blood counts (**I**). Data are represented as means ± standard deviation, with at least 4 animals per group, except for **C** and **D** where 3 mice were used. Data were tested for normal distribution by Shapiro-Wilk test; if not normally distributed, data were log-transformed. Within each sex, groups were compared using unpaired, 2-tailed *t* tests (**A**, **B**, **E**, and **F**) or 2-way ANOVA with Tukey’s multiple comparisons test (**G**–**I**). (* *P* < 0.05, ** *P* < 0.01, *** *P* < 0.001, **** *P* < 0.0001.)

To investigate the basis of EPO excess in SLC30A10 deficiency, we first measured Epo levels in our previously described *Slc30a10^–/–^* mouse model (6). Serum Epo levels were increased in female and male mutant mice (Figure 1A). To identify a potential source of excess Epo, *Epo* RNA levels were assessed and found to be decreased in kidneys yet increased in livers of *Slc30a10^–/–^* mice (Figure 1B). This suggested that the liver is the source of excess EPO in SLC30A10 deficiency. We attempted to measure *EPO* RNA levels in liver biopsies from patients with SLC30A10 mutations (21, 22), but biopsies yielded inadequate quality RNA for analysis. Given that EPO is a hypoxia-regulated gene, we next used PCR arrays to assess expression of other hypoxia-regulated genes. We found 24% of 93 hypoxia-regulated genes, including *Epo*, serpin family E member 1 (*Serpine1*), hexokinase 2 (*Hk2*), and annexin A2 (*Anxa2*), were upregulated at least 2-fold in mutant livers (Figure 1, C and D). Upregulation of *Serpine1*, *Hk2*, and *Anxa2*, the top 3 upregulated genes after *Epo*, was validated by qPCR in *Slc30a10^–/–^* livers (Figure 1E).

We next determined if metal excess contributes to Epo excess in mutant mice. Excess cobalt can stimulate hypoxia-regulated gene expression in the absence of hypoxia (23). We measured liver cobalt levels in *Slc30a10^–/–^* mice, but levels were decreased in female mice and unchanged in male mice (Figure 1F). This suggested that cobalt excess was not driving Epo excess in *Slc30a10^–/–^* mice. To determine if Mn excess contributed to Epo excess and polycythemia in mutant mice, we weaned *Slc30a10^–/–^* mice onto Mn-deficient diets and measured liver Mn levels, liver *Epo* RNA levels, and red blood cell (RBC) counts in 6-week-old mice. Liver Mn and *Epo* RNA levels and RBC counts were decreased in mutant mice weaned onto Mn-deficient diets (Figure 1, G–I). This suggested that Mn excess plays a causal role in Epo excess and polycythemia in *Slc30a10^–/–^* mice.

### Slc39a14 deficiency corrects liver Epo excess and polycythemia in Slc30a10^–/–^ mice.

**Figure 2:**
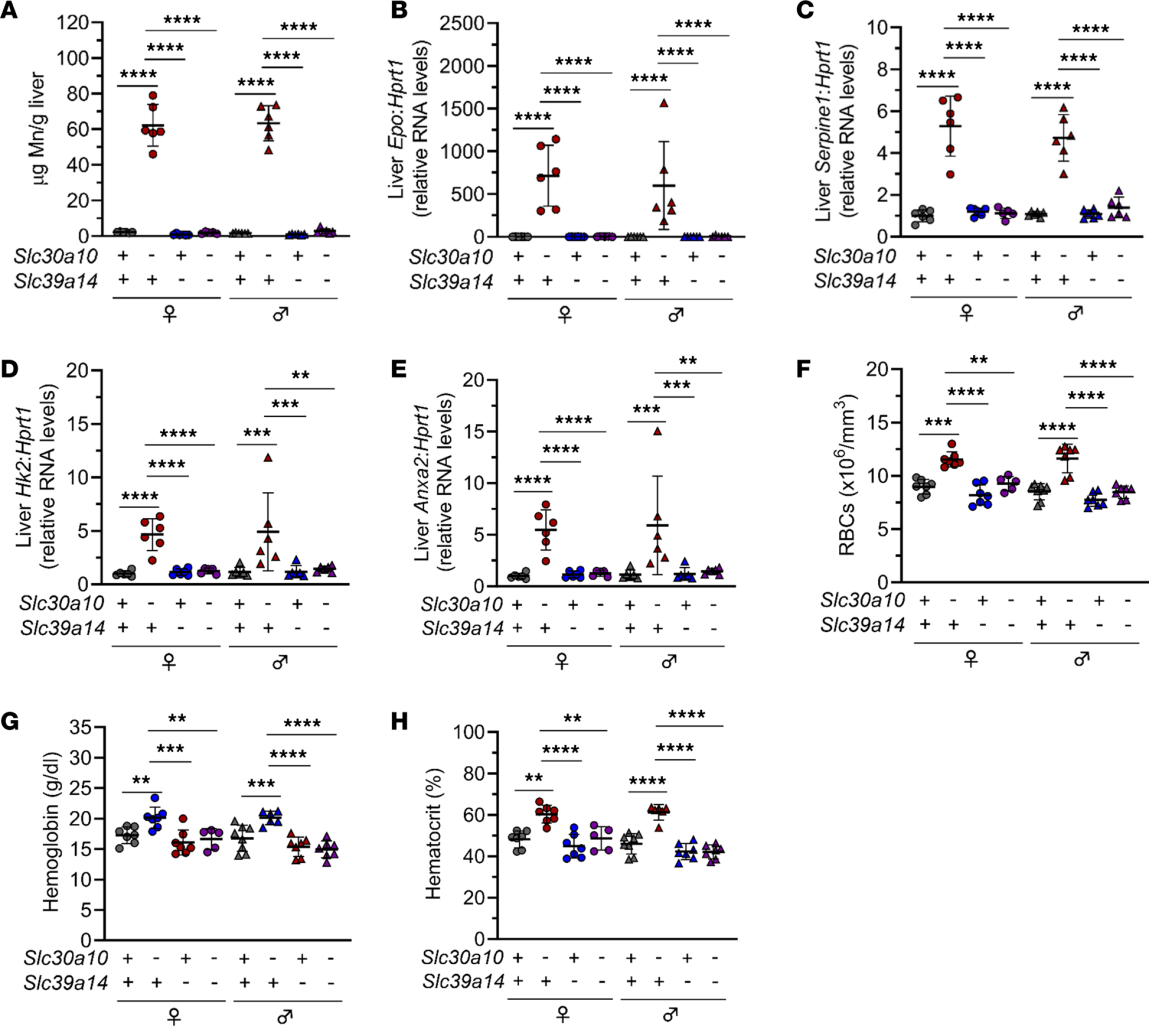
Slc39a14 deficiency corrects liver *Epo* excess and polycythemia in *Slc30a10^–/–^* mice. Five-week-old *Slc30a10 Slc39a14* mice were analyzed for liver Mn levels by ICP-OES (**A**); liver *Epo* (**B**), *Serpine1* (**C**), *Hk2* (**D**), and *Anxa2* (**E**) RNA levels by qPCR; and RBC counts (**F**), hemoglobin levels (**G**), and hematocrits (**H**) by complete blood counts. Data are represented as means ± standard deviation, with at least 5 animals per group. Data were tested for normal distribution by Shapiro-Wilk test; if not normally distributed, data were log-transformed. Groups within each sex were compared by 2-way ANOVA with Tukey’s multiple comparisons test. (** *P* < 0.01, *** *P* < 0.001, **** *P* < 0.0001.)

To further investigate the basis of EPO excess and polycythemia in SLC30A10 deficiency, we interrogated SLC39A14. Essential for gastrointestinal Mn excretion, SLC39A14 imports Mn from blood into hepatocytes and enterocytes. Both SLC30A10 and SLC39A14 deficiency cause Mn excess and neurologic disease, but SLC39A14 deficiency is not associated with liver disease, liver Mn excess, or polycythemia. Given the absence of liver Mn excess and polycythemia in SLC39A14 deficiency, we determined the impact of Slc39a14 deficiency on *Slc30a10^–/–^* mice by generating and characterizing Slc30a10- and Slc39a14-deficient mice (*Slc30a10^–/–^ Slc39a14^–/–^*) (Supplemental Figure 1, A and B; supplemental material available online with this article; https://doi.org/10.1172/jci.insight.169738DS1). Slc39a14 deficiency normalized liver Mn levels, indicating that Slc39a14 is essential for Mn excess in Slc30a10 deficiency as previously shown (24) (Figure 2A). Slc39a14 deficiency also normalized liver *Epo*, *Serpine1*, *Hk2*, and *Anxa2* RNA levels; RBC counts; hemoglobin levels; and hematocrits in *Slc30a10^–/–^* mice (Figure 2, B–H). This indicated that Slc39a14 is also essential for aberrant expression of several hypoxia-regulated genes and polycythemia in Slc30a10 deficiency. We next assessed *Epo* RNA and Mn levels in kidneys. In contrast to its impact on liver *Epo* RNA and Mn levels, Slc39a14 deficiency had no impact on kidney *Epo* RNA levels and increased kidney Mn levels in *Slc30a10^–/–^* mice (Supplemental Figure 1, C and D). Finally, we measured Mn levels in other extrahepatic sites. Slc39a14 deficiency did not change blood or pancreas Mn levels but did increase bone, brain, and heart Mn levels and decrease small and large intestine Mn levels in *Slc30a10^–/–^* mice (Supplemental Figure 1, E–K). Overall, characterization of mice with Slc39a14 and Slc30a10 deficiency indicated that Slc39a14 is essential for development of liver Mn excess, *Epo* excess, and polycythemia in *Slc30a10^–/–^* mice. This suggested that SLC39A14-dependent import of Mn into liver drives EPO excess and polycythemia in SLC30A10 deficiency. However, given that Slc39a14 deficiency also impacted Mn levels in extrahepatic tissues, we could not formally exclude a role for SLC39A14 in those other tissues in determining EPO excess and polycythemia.

### Hif1a and Hif2a levels are increased in livers of Slc30a10^–/–^ mice.

**Figure 3:**
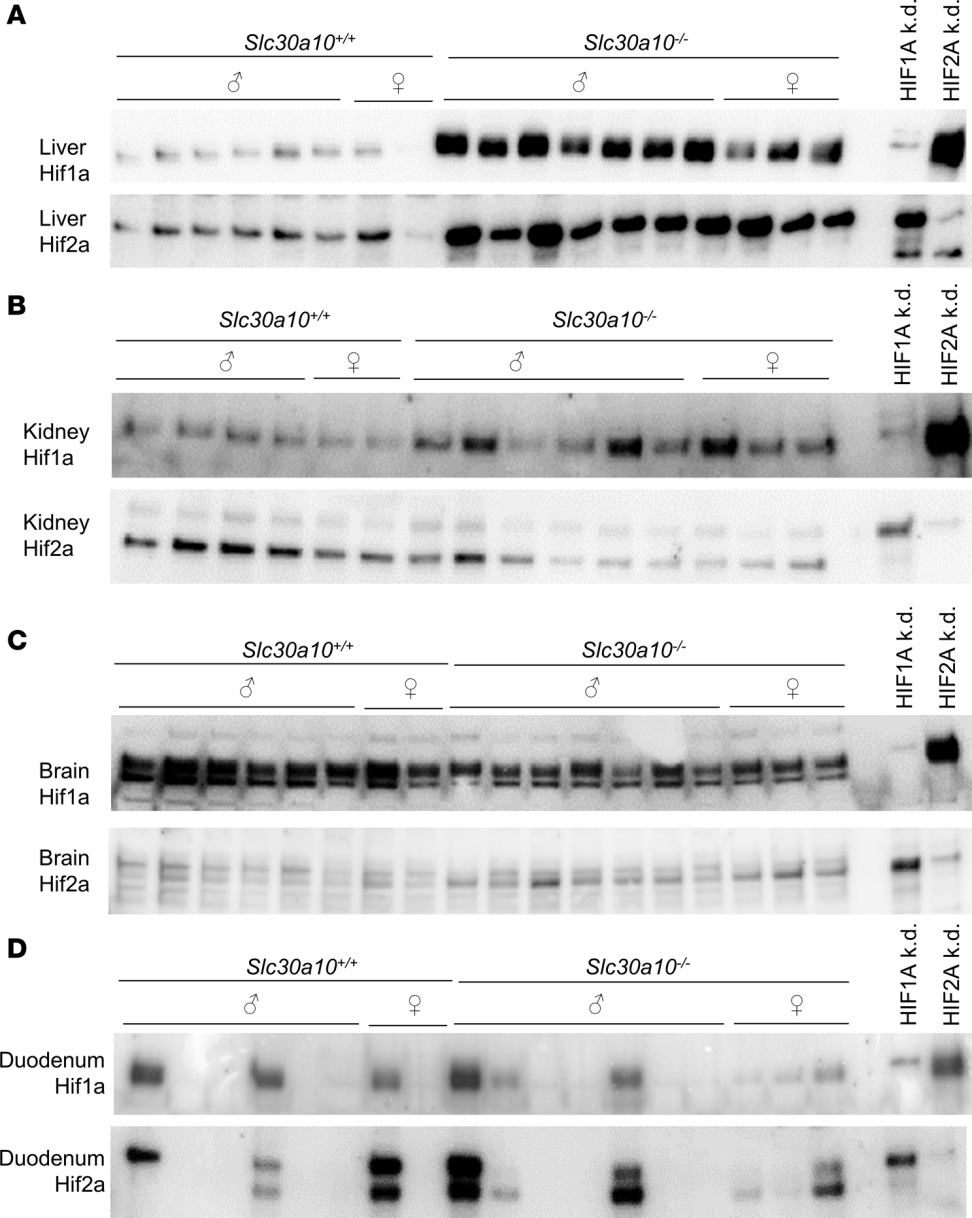
Hif1a and Hif2a protein levels are increased in livers of *Slc30a10^–/–^* mice. Two-month-old *Slc30a10* mice were analyzed for Hif1a and Hif2a protein levels by denaturing, reducing immunoblots of nuclear preps from liver (**A**), kidney (**B**), brain (**C**), and duodenum (**D**). Two rightmost lanes represent nuclear preps from Hep3B cell lines transfected with *HIF1A* (HIF1A k.d.) or *HIF2a* (HIF2A k.d.) siRNA, then treated with 100 μM MnCl_2_. Total protein images are shown in Supplemental Figure 2.

*EPO* is the canonical hypoxia-regulated gene, and HIFs are essential mediators of the cellular response to hypoxia. To determine if Hifs mediate Epo excess in *Slc30a10^–/–^* mice, we first measured Hif1a and Hif2a protein levels in nuclear extracts from multiple tissues in *Slc30a10^–/–^* mice. Hif1a and Hif2a levels were both increased in mutant livers relative to wild-type livers (Figure 3A). Hif1a levels were increased and Hif2a levels decreased in mutant kidneys (Figure 3B). Hif1a levels were decreased and Hif2a levels increased in mutant brains (Figure 3C). Hif1a and Hif2a levels did not show a consistent trend within or between genotypes for duodenum (Figure 3D). (Total protein images for all blots are shown in Supplemental Figure 2.) While these results do need to be interpreted in light of the fact that they represent whole organ homogenates, we decided to focus on liver Hif1a and Hif2a for the remainder of this study for 2 reasons. First, *Epo* RNA levels were increased in mutant livers. Second, only in mutant livers were Hif1a and Hif2a protein levels both increased.

### Hif2a antisense oligonucleotides decrease liver Epo RNA levels in Slc30a10^–/–^ mice.

**Figure 4:**
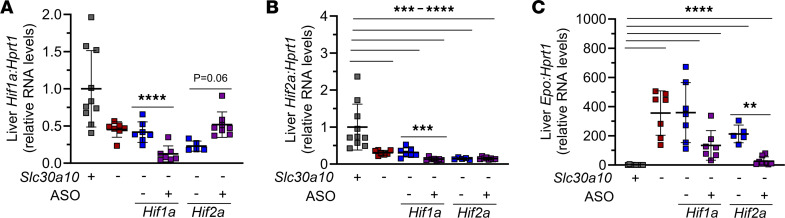
*Hif2a* ASOs decrease liver *Epo* RNA levels in *Slc30a10^–/–^* mice. Weanling *Slc30a10^+/+^* mice were treated with saline and *Slc30a10^–/–^* mice with GalNAc-conjugated control, *Hif1a* ASO, or *Hif2a* ASO twice a week for 3 weeks. Mice were then analyzed for liver *Hif1a* (**A**), *Hif2a* (**B**), and *Epo* (**C**) RNA levels by qPCR. Data are represented as means ± standard deviation, with at least 5 animals per group. Data were tested for normal distribution by Shapiro-Wilk test; if not normally distributed, data were log-transformed. Groups were compared using 2-way ANOVA with Tukey’s multiple comparisons test. (** *P* < 0.01, *** *P* < 0.001, **** *P* < 0.0001.)

To determine if Hif1 and/or Hif2 contribute to Epo excess and polycythemia in *Slc30a10^–/–^* mice, we applied 2 approaches, 1 short-term and the other long-term. In the short-term approach, we treated 3-week-old mutant mice with GalNAc-conjugated control, *Hif1a* antisense oligonucleotide (ASO), or *Hif2a* ASO for 3 weeks. We measured liver *Slc30a10* RNA levels and observed no impact of ASO treatment on *Slc30a10* RNA levels (Supplemental Figure 3A). We next assessed liver *Hif* RNA levels. Surprisingly, liver *Hif1a* and *Hif2a* RNA levels were decreased in untreated *Slc30a10^–/–^* mice (although the decrease in liver *Hif1* RNA levels in mutant mice did not reach significance) (Figure 4, A and B). *Hif1a* ASOs decreased liver *Hif1a* and *Hif2a* RNA levels beyond the decrease observed in untreated *Slc30a10^–/–^* mice. In contrast, *Hif2a* ASOs increased liver *Hif1a* RNA levels, while they had no impact on liver *Hif2a* RNA levels possibly because *Hif2a* RNA levels were already low in untreated *Slc30a10^–/–^* mice. Measurement of kidney *Hif1a* and *Hif2a* RNA levels revealed no changes in ASO-treated mice (data not shown). We next assessed the impact of ASOs on *Epo* levels and RBC parameters. *Hif2a* ASOs, but not *Hif1a* ASOs, decreased liver *Epo* RNA levels, while neither ASO impacted kidney *Epo* RNA levels or RBC parameters (Figure 4C and Supplemental Figure 3, B–E). (The decrease in liver *Epo* RNA levels in *Hif1a* ASO–treated mice was not significant.) We also observed that *Hif2a* ASOs increased body mass in *Slc30a10^–/–^* mice (Supplemental Figure 3F). Finally, we noted that *Hif1a* ASOs decreased bile flow rates while neither ASO altered bile or liver Mn levels (Supplemental Figure 3, G–I). (Despite severe Mn excess, bile Mn levels were not elevated in *Slc30a10^–/–^* mice, which we attribute to Slc30a10’s essential role in hepatobiliary Mn excretion.) Taken together, these data suggested that Hif2a, but not Hif1a, contributes to Epo excess in *Slc30a10^–/–^* mice. Given that we only treated mice with ASOs for 3 weeks, we did not anticipate a change in RBC parameters. To explore the long-term impact of Hif deficiency on phenotypes in mutant mice, we applied another approach, which is described next.

### Hepatocyte Hif1a deficiency has minimal impact on multiple phenotypes in Slc30a10^–/–^ mice.

**Figure 5:**
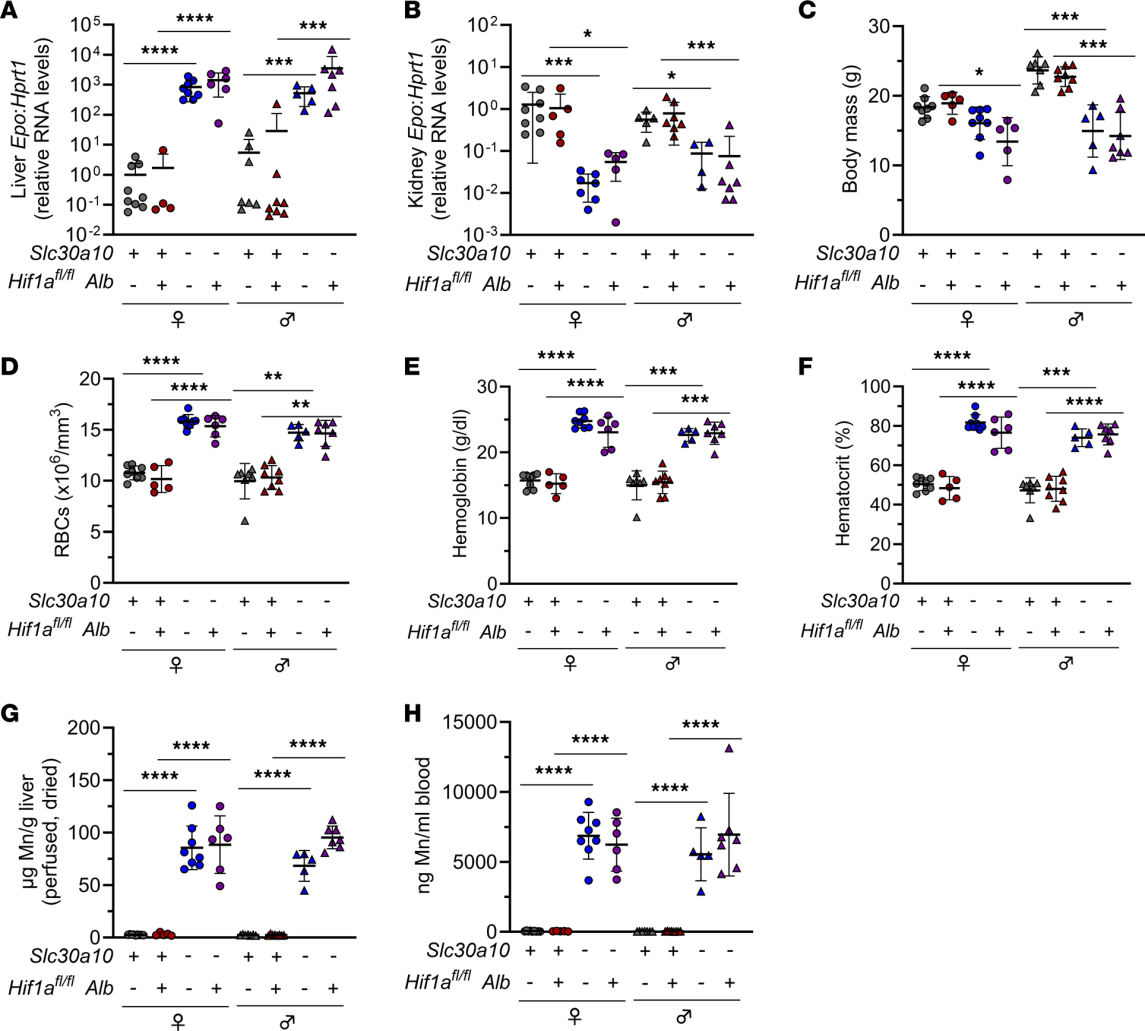
Hepatocyte Hif1a deficiency does not affect *Epo* levels or polycythemia in *Slc30a10^–/–^* mice. Two-month-old *Slc30a10 Hif1a^fl/fl^ ± Alb* mice were analyzed for liver (**A**) and kidney (**B**) *Epo* RNA levels by qPCR; body mass (**C**); RBC counts (**D**), hemoglobin levels (**E**), and hematocrits (**F**) by complete blood counts; liver Mn levels by ICP-OES (**G**); and blood Mn levels by GFAAS (**H**). Data are represented as means ± standard deviation, with at least 5 animals per group. Data were tested for normal distribution by Shapiro-Wilk test; if not normally distributed, data were log-transformed. Groups were compared using 2-way ANOVA with Tukey’s multiple comparisons test. (* *P* < 0.05, ** *P* < 0.01, *** *P* < 0.001, **** *P* < 0.0001.)

In our long-term approach to determine if Hif1 and/or Hif2 mediate Epo excess in *Slc30a10^–/–^* mice, we generated 2-month-old *Slc30a10^–/–^* mice with hepatocyte *Hif1a* or *Hif2a* deficiency using an albumin promoter-driven Cre recombinase transgene (*Alb*). We first characterized *Slc30a10 Hif1a Alb* mice. Hepatocyte Hif1a deficiency had no impact on liver *Slc30a10* RNA levels (Supplemental Figure 4A). It also decreased liver *Hif1a* RNA and Hif1a protein levels but had no effect on liver *Hif2a* RNA or Hif2a protein levels or kidney *Hif1a* or *Hif2a* RNA levels (Supplemental Figure 4, B–E; data not shown). Hepatocyte Hif1a deficiency did not impact the increased liver *Epo* RNA levels, decreased kidney *Epo* RNA levels, decreased body mass, or aberrant RBC parameters in *Slc30a10^–/–^* mice (Figure 5, A–F). It also had no impact on bile flow rates or bile, liver, or blood Mn levels in *Slc30a10^–/–^* mice (data not shown; Figure 5, G and H).

**Figure 6:**
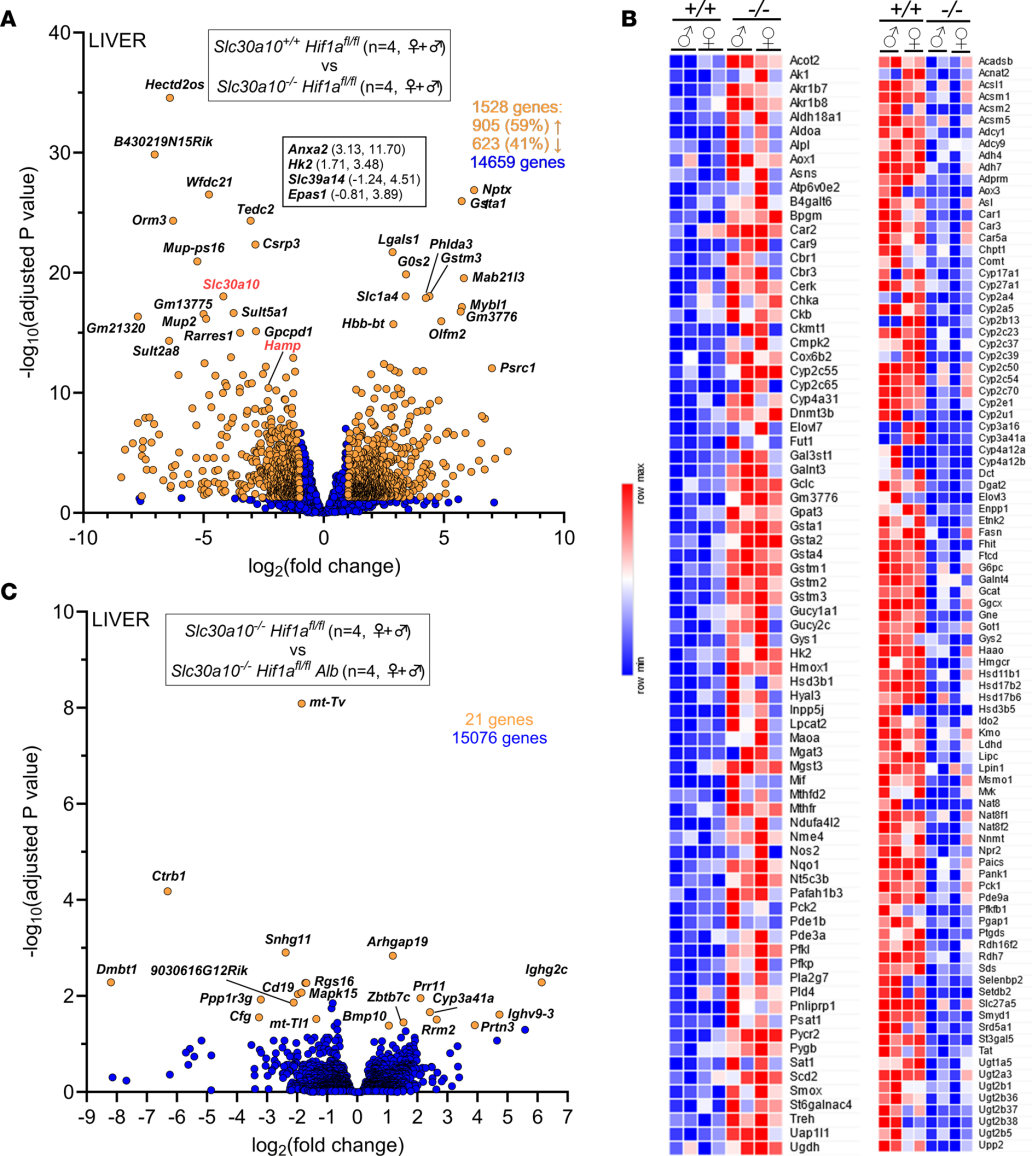
Hepatic Hif1a deficiency has minimal impact on hepatic gene expression in *Slc30a10^–/–^* mice. (**A** and **B**) Bulk RNA-Seq was performed on livers from 2-month-old *Slc30a10^–/–^ Hif1a^fl/fl^* and *Slc30a10^+/+^ Hif1a^fl/fl^* mice. Two females and 2 males were analyzed per genotype. Shown are volcano plot (**A**) and heatmap of genes aligning with metabolic pathways (**B**). (**C**) Bulk RNA-Seq was performed on livers from 2-month-old *Slc30a10^–/–^ Hif1a^fl/fl^* *Alb* and *Slc30a10^–/–^ Hif1a^fl/fl^* mice. Two females and 2 males were analyzed per genotype. Volcano plot is shown. In volcano plot, differentially expressed genes [adjusted *P* < 0.05 and absolute value of log_2_(fold-change) > 1] are shown as light orange points with gene names shown adjacent as space permitted; nondifferentially expressed genes are shown as blue points; *x*,*y* coordinates of additional genes of interest are shown in smaller box. Genes with log_2_(fold-change) < 0 are more abundantly expressed in first group listed in box at top of plot; genes with log_2_(fold-change) > 0 are more abundantly expressed in second group listed.

To further interrogate the role of hepatic Hif1a in Slc30a10 deficiency, we next determined if hepatic Hif1a deficiency impacted differential gene expression in *Slc30a10^–/–^* mice using bulk RNA-Seq. To do this, we first established the impact of Slc30a10 deficiency on hepatic gene expression by comparing *Slc30a10^–/–^* *Hif1a^fl/fl^* and *Slc30a10^+/+^* *Hif1a^fl/fl^* livers. *Slc30a10^+/+^* *Hif1a^fl/fl^* samples grouped by sex, while *Slc30a10^–/–^* *Hif1a^fl/fl^* samples clustered together (Supplemental Figure 5, A and B). Out of 14,659 genes, 1,528 were differentially expressed in Slc30a10 deficiency (Figure 6A). Included in these genes were *Slc30a10* (downregulated as expected in *Slc30a10^–/–^* mice), *Hk2*, and *Anxa2* (upregulated, as shown earlier in Figure 1), and hepcidin (*Hamp*; downregulated; discussed below), but not *Epo* (also discussed below). Differentially expressed genes aligned with a variety of pathways, with the largest number of genes aligning with metabolic pathways (Supplemental Figure 5C and Figure 6B). (We also assessed if hepatic Hif1a deficiency impacted gene expression in wild-type mice by comparing *Slc30a10^+/+^* *Hif1a^fl/fl^ Alb* and *Slc30a10^+/+^* *Hif1a^fl/fl^* livers. This identified only 20 differentially expressed genes out of 13,791 total detected; data not shown.)

After identifying liver genes differentially expressed in Slc30a10 deficiency, we next determined which genes were differentially expressed in a Hif1a-dependent manner by comparing *Slc30a10^–/–^ Hif1a^fl/fl^ Alb* and *Slc30a10^–/–^ Hif1a^fl/fl^* livers. Samples did not group by genotype or sex (Supplemental Figure 5, D and E). Only 21 genes were differentially expressed out of 15,076 detected (Figure 6C). Taken together, the above results indicated that hepatic Hif1a deficiency had minimal impact on Epo excess, polycythemia, Mn levels, and hepatic differential gene expression in *Slc30a10^–/–^* mice.

### Hepatocyte Hif2a deficiency corrects Epo excess and polycythemia and attenuates Mn levels and aberrant liver gene expression in Slc30a10^–/–^ mice.

**Figure 7:**
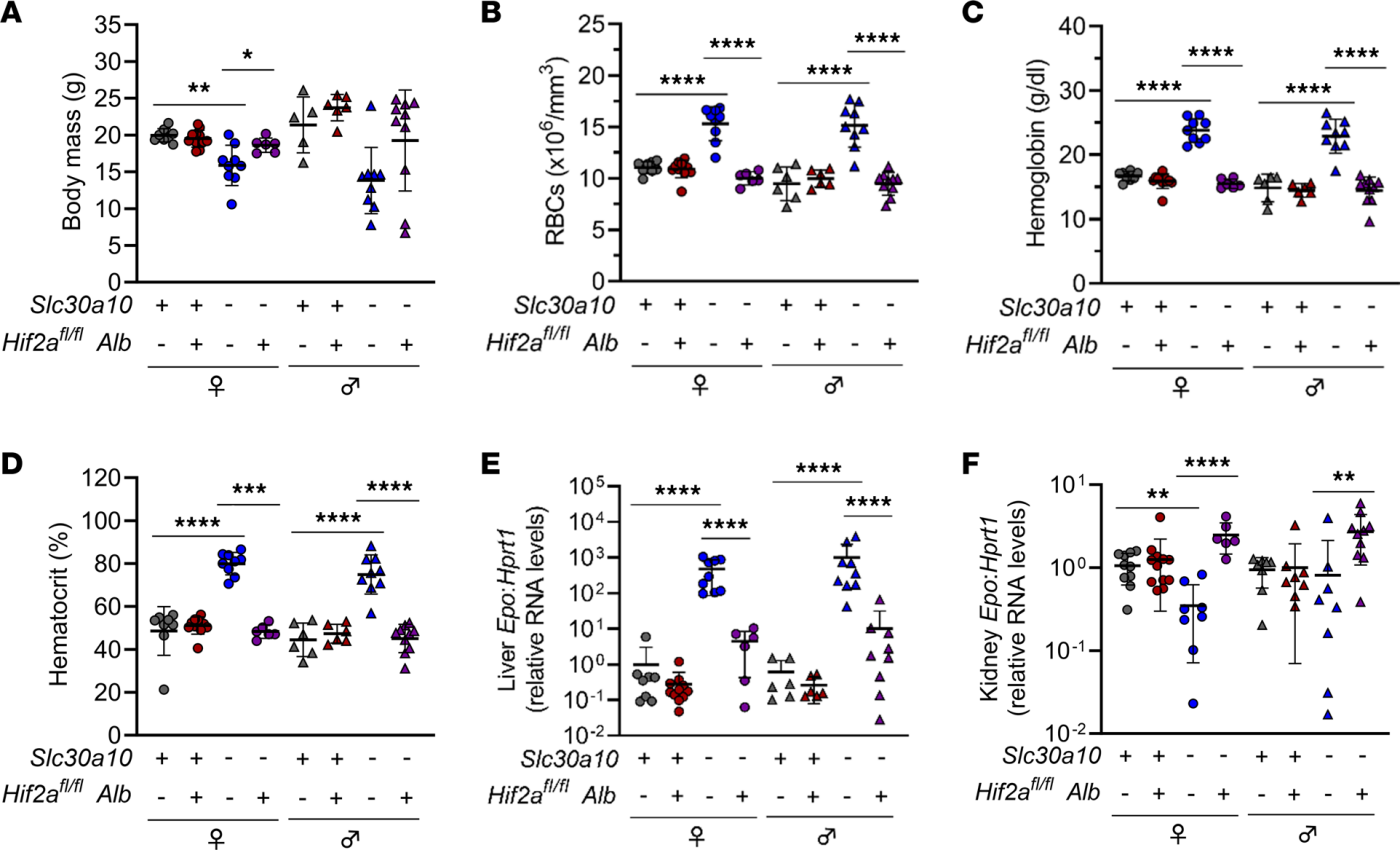
Hepatocyte Hif2a deficiency corrects liver *Epo* excess and polycythemia in *Slc30a10^–/–^* mice. Two-month-old *Slc30a10 Hif2a^fl/fl^* ± *Alb* mice were analyzed for body mass (**A**); RBC counts (**B**), hemoglobin levels (**C**), and hematocrits (**D**) by complete blood counts; and liver (**E**) and kidney (**F**) *Epo* RNA levels by qPCR. Data are represented as means ± standard deviation, with at least 5 animals per group. Data were tested for normal distribution by Shapiro-Wilk test; if not normally distributed, data were log-transformed. Within each sex, groups were compared using 2-way ANOVA with Tukey’s multiple comparisons test. (* *P* < 0.05, ** *P* < 0.01, *** *P* < 0.001, **** *P* < 0.0001.)

**Figure 8:**
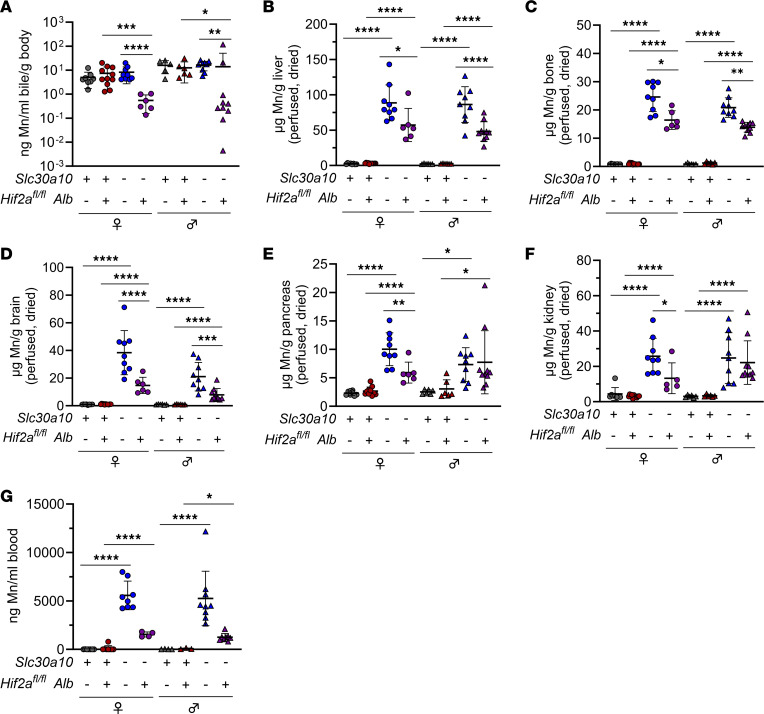
Hepatocyte Hif2a deficiency attenuates Mn excess in *Slc30a10^–/–^* mice. Two-month-old *Slc30a10 Hif2a^fl/fl^* ± *Alb* mice were analyzed for bile Mn levels by GFAAS (**A**); liver (**B**), bone (**C**), brain (**D**), pancreas (**E**), and kidney (**F**) Mn levels by ICP-OES; and blood Mn levels by GFAAS (**G**). Data are represented as means ± standard deviation, with at least 5 animals per group. Data were tested for normal distribution by Shapiro-Wilk test; if not normally distributed, data were log-transformed. Within each sex, groups were compared using 2-way ANOVA with Tukey’s multiple comparisons test. (* *P* < 0.05, ** *P* < 0.01, *** *P* < 0.001, **** *P* < 0.0001.)

To further explore the impact of Hif proteins on Slc30a10 deficiency, we next generated and characterized *Slc30a10 Hif2a Alb* mice. Hepatocyte Hif2a deficiency had no impact on liver *Slc30a10* or *Hif1a* RNA levels but did decrease liver *Hif2a* RNA levels and liver Hif2a and Hif1a protein levels (Supplemental Figure 6). Hepatocyte Hif2a deficiency also increased body mass in female *Slc30a10^–/–^* mice (and insignificantly in male mutant mice) and normalized RBC parameters in all *Slc30a10^–/–^* mice (Figure 7, A–D). In contrast with hepatocyte Hif1a deficiency, hepatocyte Hif2a deficiency decreased liver *Epo* RNA levels and increased kidney *Epo* RNA levels in *Slc30a10^–/–^* mice (Figure 7, E and F). We also assessed bile, blood, and tissue Mn levels in *Slc30a10 Hif2a* mice. Hepatocyte Hif2a deficiency had no impact on bile flow rates but did decrease Mn levels in bile, liver, bone, brain, pancreas (in females), kidney (in females), and blood in *Slc30a10^–/–^* mice, though decreases in blood were not significant (data not shown; Figure 8).

We next considered the extent to which correction of polycythemia by hepatic Hif2a deficiency in *Slc30a10^–/–^* mice contributed to decreased tissue Mn levels shown above. To clear blood from tissues so that tissue Mn levels would reflect parenchymal but not blood Mn, *Slc30a10 Hif1a* and *Slc30a10 Hif2a* mice were perfused with saline prior to tissue harvest, and tissues were dried before metal measurements. To assess the efficacy of perfusion on clearing blood from tissues, we focused on tissue iron levels. We previously demonstrated that *Slc30a10^–/–^* mice (without perfusion prior to tissue harvest) have increased total iron levels in liver (data regraphed here for reference in Supplemental Figure 7A) (6). However, liver nonheme iron levels did not differ between *Slc30a10^+/+^* and *Slc30a10^–/–^* mice (Supplemental Figure 7B). This indicated that the increased total iron levels largely reflected RBC hemoglobin, not parenchymal iron, in the liver. We next analyzed total and nonheme iron levels in livers from perfused *Slc30a10 Hif1a* and *Slc30a10 Hif2a* mice. Despite the presence of polycythemia in *Slc30a10^–/–^ Hif1a^fl/fl^* and *Slc30a10^–/–^ Hif1a^fl/fl^ Alb* mice, neither total nor nonheme iron levels were increased in livers from these mice relative to their *Slc30a10^+/+^* counterparts (Supplemental Figure 7, C and D). Similarly, despite the presence of polycythemia in *Slc30a10^–/–^ Hif2a^fl/fl^* mice, neither total nor nonheme iron levels were increased in livers from these mice relative to *Slc30a10^+/+^ Hif2a^fl/fl^* mice (Supplemental Figure 7, E and F). These findings suggested that perfusion was effective at clearing blood from livers in polycythemic mice. (Nonheme iron levels were decreased in *Slc30a10^–/–^ Hif1a^fl/fl^ Alb* livers relative to *Slc30a10^–/–^ Hif1a^fl/fl^* livers, while total and nonheme iron levels were increased in *Slc30a10^–/–^ Hif2a^fl/fl^ Alb* livers relative to *Slc30a10^+/+^ Hif2a^fl/fl^ Alb* livers in female mice — the relevance of this is not clear at this time.) In our previously published study, we also observed that total iron levels were increased in pancreas, kidney, and brain in *Slc30a10^–/–^* mice (data regraphed here for reference in Supplemental Figure 7, G–I) (6). Total iron levels in pancreas, kidney, or brain (males only) did not differ by genotype for *Slc30a10 Hif2a* mice, suggesting that perfusion was effective at clearing blood from these tissues (Supplemental Figure 7, J–L). (Total iron levels were increased in *Slc30a10^–/–^ Hif2a^fl/fl^* brains relative to *Slc30a10^+/+^ Hif2a^fl/fl^* brains for female mice; Supplemental Figure 7L.) Overall, these analyses indicated that the decrease in tissue Mn levels in *Slc30a10^–/–^ Hif2a^fl/fl^ Alb* mice was due to a decrease in parenchymal Mn content, not in correction of polycythemia.

We next assessed the impact of Hif2a on differential gene expression in Slc30a10 deficiency. We first identified genes differentially expressed in Slc30a10 deficiency by comparing *Slc30a10^–/–^ Hif2a^fl/fl^* and *Slc30a10^+/+^ Hif2a^fl/fl^* livers. Samples segregated by *Slc30a10* genotype and sex (Supplemental Figure 8, A and B). Out of 14,873 genes, 1,712, including *Slc30a10* (downregulated in *Slc30a10^–/–^* livers), *Serpine1*, *Hk2*, *Anxa2* (all upregulated), and *Hamp* (downregulated), were differentially expressed in *Slc30a10^–/–^ Hif2a^fl/fl^* livers relative to *Slc30a10^+/+^ Hif2a^fl/fl^* livers (Supplemental Figure 8C). Differentially expressed genes aligned with a variety of pathways, with the largest number of genes aligning with metabolic pathways (Supplemental Figure 8, D and E). (We also assessed if hepatic Hif2a deficiency impacted gene expression in wild-type mice by comparing *Slc30a10^+/+^* *Hif2a^fl/fl^ Alb* and *Slc30a10^+/+^* *Hif2a^fl/fl^* livers. This identified only 1 differentially expressed gene out of 13,801 total detected; data not shown.)

**Figure 9:**
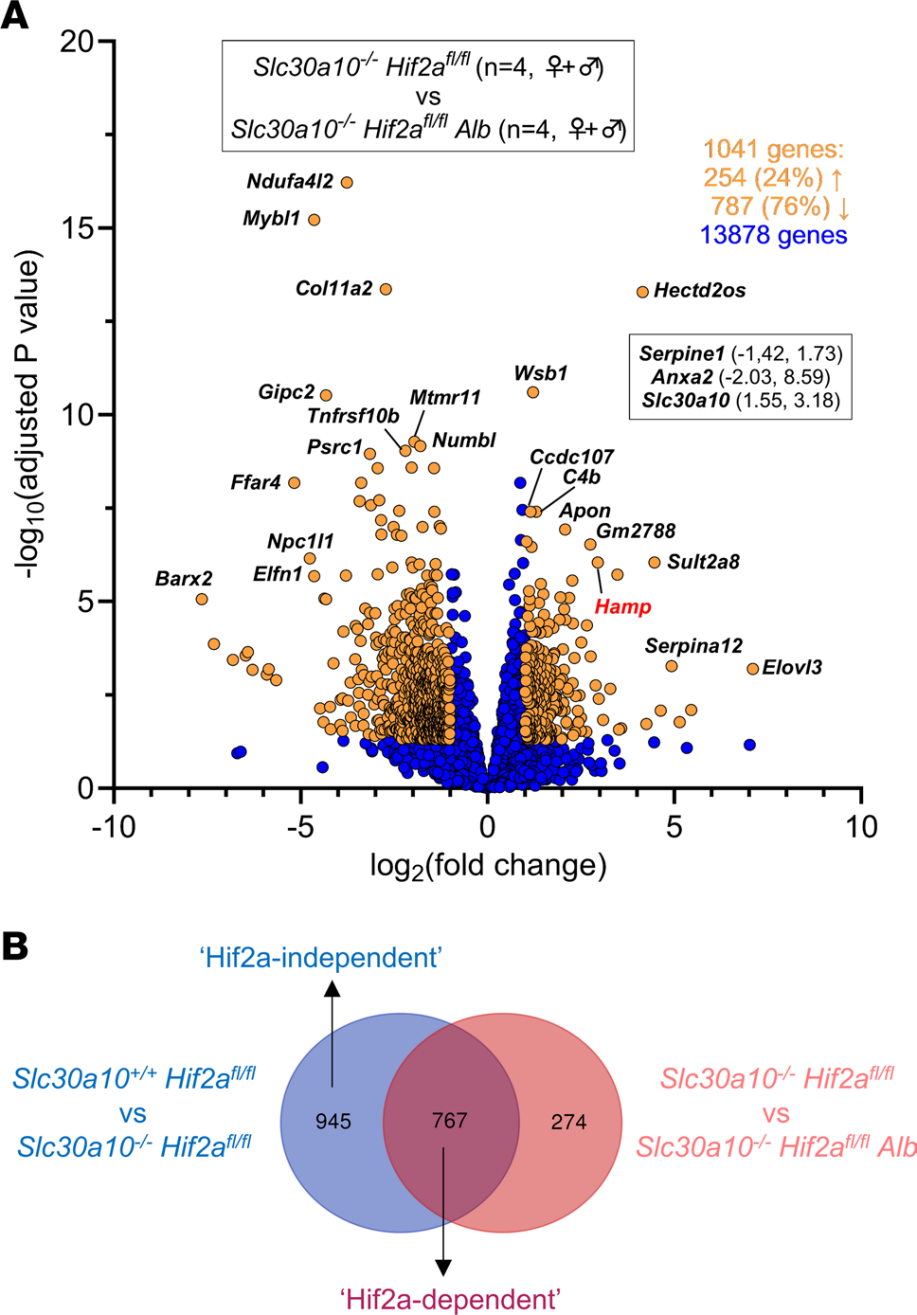
Hepatocyte Hif2a deficiency attenuates differential gene expression in livers of *Slc30a10^–/–^* mice. Bulk RNA-Seq was performed on livers from 2-month-old *Slc30a10^–/–^ Hif2a^fl/fl^ Alb* and *Slc30a10^–/–^ Hif2a^fl/fl^* mice. Two females and 2 males were analyzed per genotype. (**A**) Volcano plot. Differentially expressed genes [adjusted *P* < 0.05 and absolute value of log_2_(fold-change) > 1] are shown as light orange points with gene names shown adjacent as space permitted; nondifferentially expressed genes are shown as blue points; *x*,*y* coordinates of additional genes of interest are shown in smaller box. Genes with log_2_(fold-change) < 0 are more abundantly expressed in first group listed in box at top of plot; genes with log_2_(fold-change) > 0 are more abundantly expressed in second group listed. (**B**) Genes differentially expressed with Slc30a10 deficiency and genes differentially expressed with hepatic Hif2a deficiency in Slc30a10-deficient mice were compared with Venn diagrams and gene pathway enrichment.

To establish the impact of hepatic Hif2a deficiency on gene expression in Slc30a10 deficiency, we compared *Slc30a10^–/–^ Hif2a^fl/fl^ Alb* and *Slc30a10^–/–^ Hif2a^fl/fl^* livers. Samples clustered by *Slc30a10* genotype and sex (Supplemental Figure 9). Out of 14,919 genes, 1,041, including *Anxa2*, *Serpine1* (both downregulated with Hif2a deficiency in *Slc30a10^–/–^* livers), and *Hamp* (upregulated), were differentially expressed in *Slc30a10^–/–^ Hif2a^fl/fl^ Alb* versus *Slc30a10^–/–^ Hif2a^fl/fl^* livers (Figure 9A). We next assigned genes differentially expressed in *Slc30a10 Hif2a* mice to 3 groups. The first group, termed “Hif2a-independent,” consisted of 945 genes differentially expressed in Slc30a10 deficiency but not impacted by hepatic Hif2a deficiency (Figure 9B). These genes aligned with a variety of pathways, with the largest number of genes aligning with metabolic pathways (Supplemental Figure 10). This group represented 55% of all genes differentially expressed with Slc30a10 deficiency. Within this group, 453 genes (48% of 945 genes) were upregulated and 492 genes (52% of 945 genes) downregulated with Slc30a10 deficiency. While these genes were denoted Hif2a independent given their adjusted *P* values, expression of 98% of genes upregulated in Slc30a10 deficiency decreased with Hif2a deficiency while expression of 96% of genes downregulated in Slc30a10 deficiency increased with Hif2a deficiency, albeit insignificantly (data not shown).

The second group of *Slc30a10 Hif2a* genes, termed “Hif2a-dependent,” consisted of 767 genes differentially impacted by Slc30a10 deficiency and by hepatic Hif2a deficiency in Slc30a10-deficient mice (Figure 9B). These genes aligned with a variety of pathways (Supplemental Figure 11). Within this group, 565 genes (74% of 767 genes) were upregulated with Slc30a10 deficiency, then downregulated with Hif2a deficiency. The remaining 201 genes (26% of 767 genes) were downregulated with Slc30a10 deficiency, then upregulated with Hif2a deficiency.

The third group of *Slc30a10 Hif2a* genes were not differentially expressed in Slc30a10 deficiency but were differentially impacted by hepatic Hif2a deficiency in Slc30a10-deficient mice (Figure 9B). These 274 genes did not align with any pathway (data not shown).

We next considered the absence of *Epo* as a differentially expressed gene in several RNA-Seq analyses, despite the prominent change in liver *Epo* RNA levels in *Slc30a10^–/–^* mice. For qPCR, samples with undetectable *Epo* levels were arbitrarily assigned Ct values of 40 for calculation of fold-differences in expression (Supplemental Figure 12). However, in RNA-Seq analyses, *Epo* raw counts for most samples were below the cutoff of 10 and excluded from further analysis.

**Figure 10:**
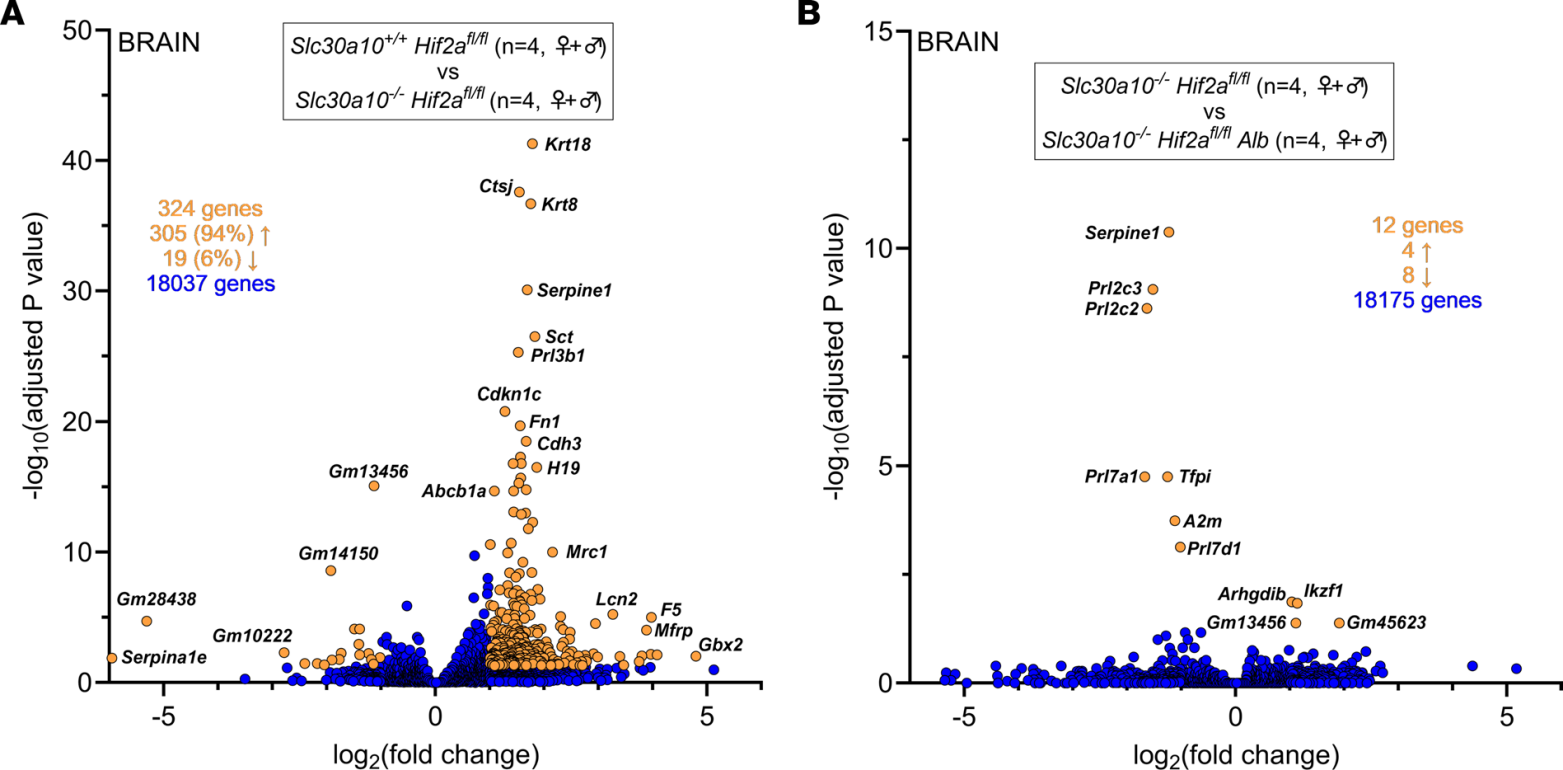
Hepatic Hif2a deficiency has minimal impact on brain gene expression in *Slc30a10^–/–^* mice. (**A**) Bulk RNA-Seq was performed on brains from 2-month-old *Slc30a10^–/–^ Hif2a^fl/fl^* and *Slc30a10^+/+^ Hif2a^fl/fl^* mice. Two females and 2 males were analyzed per genotype. Volcano plot is shown. (**B**) Bulk RNA-Seq was performed on brains from 2-month-old *Slc30a10^–/–^ Hif2a^fl/fl^ Alb* and *Slc30a10^–/–^ Hif2a^fl/fl^* mice. Two females and 2 males were analyzed per genotype. Volcano plot is shown. In volcano plots, differentially expressed genes [adjusted *P* < 0.05 and absolute value of log_2_(fold-change) > 1] are shown as light orange points with gene names shown adjacent as space permitted; nondifferentially expressed genes are shown as blue points; *x*,*y* coordinates of additional genes of interest are shown in smaller box. Genes with log_2_(fold-change) < 0 are more abundantly expressed in first group listed in box at top of plot; genes with log_2_(fold-change) > 0 are more abundantly expressed in second group listed.

Given that the brain is prominently impacted in SLC30A10 deficiency, we also investigated the impact of hepatic Hif2a deficiency on gene expression in *Slc30a10^–/–^* brains using bulk RNA-Seq. We first compared *Slc30a10^–/–^ Hif2a^fl/fl^* and *Slc30a10^+/+^ Hif2a^fl/fl^* brains. Samples did not cluster by *Slc30a10* genotype or sex (Supplemental Figure 13, A and B). Out of 18,037 genes, 324 were differentially expressed, with the majority (94%) upregulated, in Slc30a10 deficiency (Figure 10A). Genes aligned to a variety of pathways (Supplemental Figure 13, C and D). To determine if hepatic Hif2a deficiency affected differential gene expression in Slc30a10 deficiency, we compared *Slc30a10^–/–^ Hif2a^fl/fl^ Alb* and *Slc30a10^–/–^ Hif2a^fl/fl^* brains. Samples did not group by *Hif2a* genotype or sex (Supplemental Figure 13, E and F). Out of 18,175 genes, only 12 were differentially expressed (Figure 10B). Taken together, the analyses presented above indicate that hepatic Hif2a deficiency corrects Epo excess and polycythemia and attenuates tissue Mn excess. It also prominently impacts differential gene expression in the liver but not in the brain, though the latter should be interpreted in light of the fact that analyses were performed on whole brain.

### Hepatic Hif2a deficiency impacts iron homeostasis in Slc30a10^–/–^ mice.

**Figure 11:**
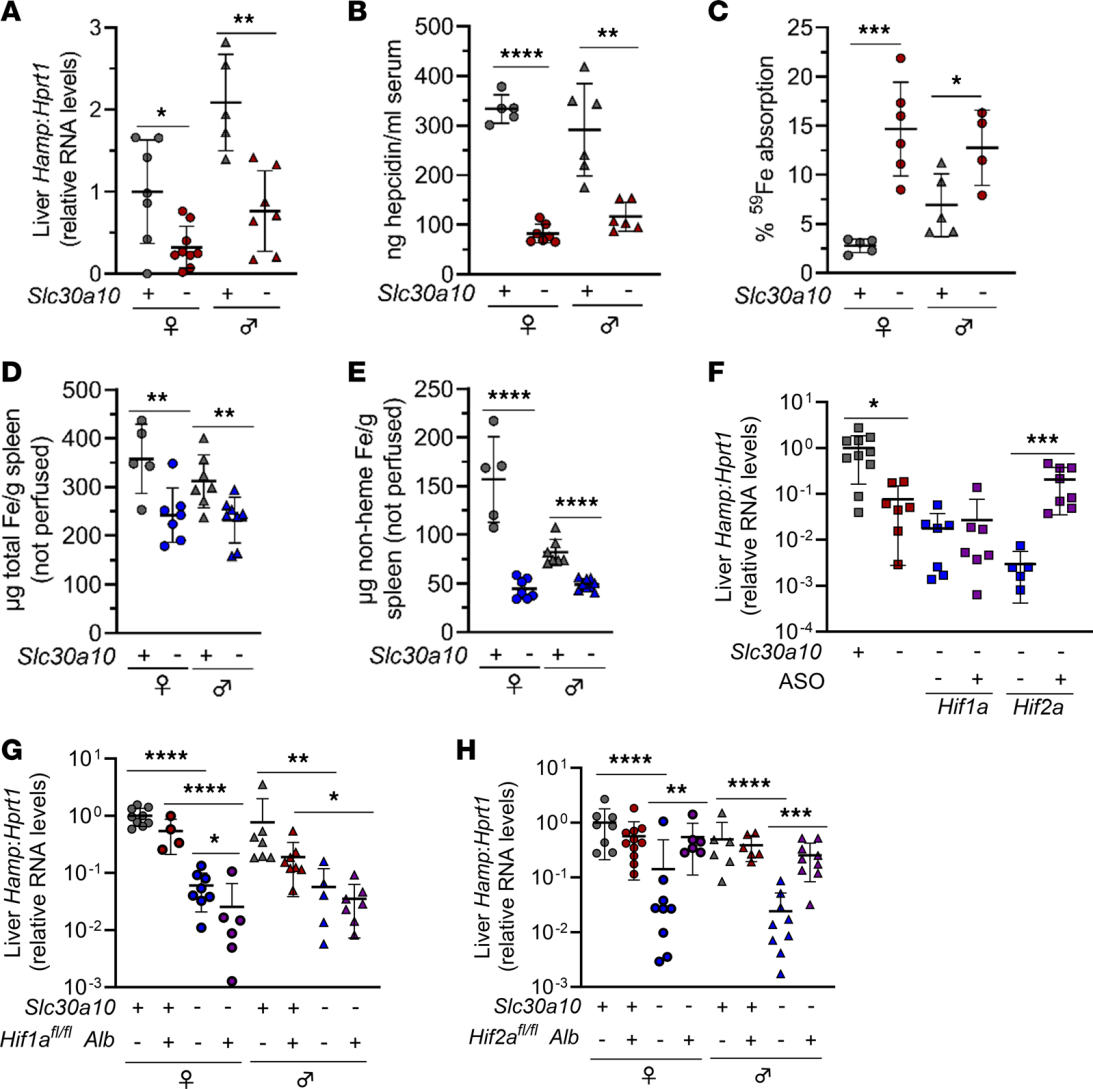
Hepatic Hif2a deficiency affects iron homeostasis in *Slc30a10^–/–^* mice. (**A**–**D**) Mice from Figure 1, A–F, were analyzed for (**A**) liver hepcidin (*Hamp*) RNA levels by qPCR; (**B**) serum hepcidin levels by ELISA; (**C**) ^59^Fe absorption by gastric gavage; (**D**) total Fe levels in spleen by ICP-OES; and (**E**) nonheme Fe levels in spleen by bathophenanthroline-based assay. (**F**–**H**) ASO-treated, *Slc30a10 Hif1a*, and *Slc30a10 Hif2a* mice were analyzed for liver *Hamp* RNA levels by qPCR. Data are represented as means ± standard deviation, with at least 4 animals per group. Data were tested for normal distribution by Shapiro-Wilk test; if not normally distributed, data were log-transformed. Within each sex, groups were compared using unpaired, 2-tailed *t* tests (**A**–**E**) or 2-way ANOVA with Tukey’s multiple comparisons test (**F**–**H**). (* *P* < 0.05, ** *P* < 0.01, *** *P* < 0.001, **** *P* < 0.0001.)

RNA-Seq analyses of *Slc30a10* livers indicated that hepcidin (*Hamp*) was downregulated with Slc30a10 deficiency in a Hif2a-dependent manner. Hepcidin is a hormone synthesized mainly by the liver that inhibits dietary iron absorption and macrophage iron export (25). It is upregulated by iron excess and inflammation and downregulated by iron deficiency and in settings of increased iron demand, such as Epo excess. We next investigated if the decreased hepcidin expression had functional impact on iron homeostasis in *Slc30a10^–/–^* mice. We first validated decreased liver hepcidin RNA levels and serum hepcidin levels in *Slc30a10^–/–^* mice (Figure 11, A and B). Consistent with hepcidin deficiency, *Slc30a10^–/–^* mice exhibited increased absorption of gavaged ^59^Fe (Figure 11C). Total and nonheme iron levels were decreased in *Slc30a10^–/–^* spleen (Figure 11, D and E), also consistent with hepcidin deficiency — since hepcidin inhibits macrophage iron export, hepcidin deficiency leads to increased iron export from red pulp macrophages, leading to decreased spleen iron levels.

Our RNA-Seq analyses presented above indicated that *Hamp* is downregulated by Slc30a10 deficiency in a Hif2a-dependent manner. To validate and further explore the link between Hif2a and hepcidin, we measured liver hepcidin RNA by qPCR in *Slc30a10^–/–^* mice with altered Hif levels. We first considered our ASO-treated *Slc30a10* cohort. We noted above that treatment of *Slc30a10^–/–^* mice with *Hif2a* ASOs, but not *Hif1a* ASOs, decreased liver *Epo* RNA levels (Figure 4C). Treatment with *Hif2a* ASOs, but not *Hif1a* ASOs, increased liver hepcidin RNA levels (Figure 11F). We also noted above that hepatic Hif2a deficiency, but not Hif1a deficiency, corrected liver *Epo* excess (Figure 5A and Figure 7E). Hepatocyte deficiency in Hif2a, but not Hif1a, increased liver hepcidin RNA levels in *Slc30a10^–/–^* mice (Figure 11, G and H). Overall, this indicated that hepatic Hif2a is essential for hepcidin downregulation in *Slc30a10^–/–^* mice.

## Discussion

SLC30A10 deficiency is the first known inherited cause of Mn excess. It is characterized by neurologic and liver dysfunction, polycythemia, and EPO excess. Neurologic and liver disease are attributed to Mn toxicity, while polycythemia is attributed to EPO excess. The cause of EPO excess in this disease has yet to be established. Based upon the data presented above, we propose the following model to explain the link between SLC30A10 deficiency and EPO excess. SLC30A10 deficiency impairs gastrointestinal Mn excretion, leading to systemic Mn excess. Excess Mn is imported into the liver (and pancreas and small and large intestines) by SLC39A14. (SLC39A14 deficiency is the second reported inherited cause of Mn excess. Unlike SLC30A10 deficiency, SLC39A14 deficiency does not produce liver dysfunction, polycythemia, or EPO excess.) Liver Mn excess leads to increased HIF2-dependent EPO expression. EPO excess results in polycythemia and suppresses liver hepcidin expression, leading to increased dietary iron absorption with the majority of excess iron consumed by erythropoiesis.

The observation that hepatic Hif2a deficiency attenuates *Epo* excess and polycythemia in *Slc30a10^–/–^* mice is consistent with a previous study showing that hepatic *Epo* expression is regulated by Hif2a, not Hif1a (26). However, our work raises several questions. First, why does hepatic Hif2a deficiency also attenuate Mn excess in *Slc30a10^–/–^* mice? One possibility is that decreased tissue Mn levels reflect correction of polycythemia, but mice were perfused with saline prior to tissue harvest. Another possibility is that hepatic Hif2a deficiency increases Mn excretion, but bile Mn levels did not increase in *Slc30a10^–/–^* mice with hepatic Hif2a deficiency. Urinary excretion is unlikely, as Mn excretion by the kidneys is traditionally viewed as minimal. Gastrointestinal Mn excretion independent of the hepatobiliary route is a possibility, but this would have to be Slc30a10 independent given that our studies were done in *Slc30a10^–/–^* mice. Another possibility is decreased dietary Mn absorption. While HIF2 and SLC30A10 have no known roles in regulating Mn absorption, *Slc30a10^–/–^* mice do exhibit hepcidin deficiency. Hepcidin inhibits dietary iron absorption by posttranslationally downregulating ferroportin, a transport protein essential for export of iron from enterocytes into blood. Several studies report that ferroportin can also transport Mn (27–33), while some do not support this (34). If hepcidin deficiency leads to increased ferroportin-dependent Mn absorption, hepatic Hif2a deficiency could decrease Mn levels by correcting hepcidin deficiency and suppressing Mn absorption. We are actively pursuing this line of investigation.

The second issue raised by our work is the minimal impact of hepatic Hif1a deficiency on *Slc30a10^–/–^* phenotypes. This observation is striking, given that Hif1a and Hif2a protein levels were both increased in nuclear extracts from *Slc30a10^–/–^* mouse livers. The abundance of Hif1a protein in mutant livers yet minimal impact of hepatic Hif1a deficiency on *Slc30a10^–/–^* phenotypes suggests that hepatic Hif1 is upregulated but its activity suppressed in Slc30a10 deficiency. One candidate that mediates selective HIF1 suppression is an asparagine hydroxylase known as factor inhibiting HIF1 (FIH) (35). FIH hydroxylates the C-terminal transactivation domain of HIF α subunits, preventing the interaction between HIFs and transcriptional coactivators. FIH hydroxylates HIF1A more efficiently than HIF2A. FIH requires iron for activity but has been shown to bind Mn in vitro, though the impact of Mn binding on FIH activity has yet to be explored (36). In addition to FIH, histone deacetylases known as sirtuins are also candidates. SIRT1 can act on both HIF1A and HIF2A with opposing effects — HIF1 deacetylation prevents recruitment of transcriptional coactivators while HIF2 deacetylation augments transcriptional activity (37, 38). However, reports on the impact of sirtuins on HIFs do not always present a consistent picture. For example, SIRT1 has also been reported to enhance HIF1 activity and repress HIF2 activity (39, 40). There are other factors known to inhibit HIF activity, but the function of these is not consistent with the prominent Hif2a and minimal Hif1a phenotype in *Slc30a10^–/–^* mice. Inhibitory PAS domain protein (IPAS) is a splice variant of HIF3A and a dominant negative regulator of both HIF1 and HIF2 (41). IPAS activity could account for Hif1 inhibition in *Slc30a10^–/–^* mice, but Hif2 should be inhibited as well. Hypoxia-associated factor, an E3 ubiquitin ligase, binds to HIF1A and HIF2A, leading to HIF1A degradation and HIF2A transactivation, but we observed increased Hif1a protein levels in livers of *Slc30a10^–/–^* mice (42).

In contexts of chronic HIF activation, increased HIF2 levels are maintained while HIF1 levels are suppressed (43–45). In our study, *Slc30a10 Hif1a* and *Slc30a10 Hif2a* mice were analyzed at 2 months of age. Mn excess in *Slc30a10^–/–^* mice develops at 14–21 days of life, and polycythemia develops at 21–28 days of life (data not shown). These observations suggest that by 2 months of age, *Slc30a10^–/–^* mice have experienced weeks of aberrant Hif activity. Given that we observed persistent upregulation of Hif1a and Hif2a in *Slc30a10^–/–^* mice, yet only hepatic Hif2a deficiency impacted gene expression in mutant livers, it appears that Slc30a10 deficiency has different effects on Hif1 versus Hif2 not commonly seen in other contexts of chronic HIF upregulation.

The third prominent topic at hand is the impact of aberrant expression of genes beyond *Epo* in livers of *Slc30a10^–/–^* mice. The majority of upregulated genes aligned with cell cycle, while the majority of downregulated genes aligned with metabolic processes. Hepatic Hif2a deficiency attenuated almost half of differential gene expression, indicating that HIF2 is a key determinant of hepatic gene expression in SLC30A10 deficiency. Given that HIF2 stimulates gene expression, we propose that upregulation of cell cycle genes in *Slc30a10^–/–^* mice is a direct result of increased Hif2 activity, while the downregulation of metabolic process genes is due to an indirect consequence of increased Hif2 activity. HIFs are well known to regulate cell proliferation (46, 47) and can impact metabolism of carbohydrates and lipids, as observed in diseases such as cancer, diabetes, and nonalcoholic fatty liver disease (48–51). Although the specific HIF-dependent pathways aberrantly expressed in *Slc30a10^–/–^* livers were not surprising given the established roles of HIFs in health and disease, it is not clear at this time how aberrant HIF2-dependent gene expression outside of EPO contributes to the pathophysiology of SLC30A10 deficiency.

Based upon the data presented above, we propose that the prominent impact of hepatic Hif2a deficiency on Mn excess, *Epo* excess, polycythemia, and hepatic gene expression warrants an investigation into the long-term treatment of *Slc30a10^–/–^* mice with agents that inhibit Hif2a expression and/or activity. One option is Hif2a ASOs as we employed above. Another option is the HIF2 inhibitor belzutifan (52), already approved for cancer treatment in patients with mutations in VHL, an E3 ubiquitin ligase essential for HIF ubiquitination and degradation. Current treatment options for SLC30A10 deficiency include chelation and oral iron supplementation, with the latter proposed to act by outcompeting dietary Mn for absorption. Our studies suggest HIF2 antagonism as a novel therapeutic option for this disease. However, one caveat that certainly deserves consideration is the minimal impact of hepatic Hif2a deficiency on differential gene expression in brains of *Slc30a10^–/–^* mice. While hepatic Hif2a deficiency did decrease brain Mn levels significantly, normalization of brain gene expression may require further lowering of brain Mn levels. We analyzed mice at 2 months of age — perhaps brain Mn levels would continue to decrease in *Slc30a10^–/–^* *Hif2a^fl/fl^ Alb* mice as they age. Additionally, we induced Hif2a deficiency using an albumin promoter-driven Cre recombinase transgene, which in our experience is fully expressed by 1 month of age. Given that *Slc30a10^–/–^* mice develop Mn excess between 2 and 3 weeks of life (data not shown), hepatic Hif2a inactivation may have occurred after sufficient Mn had already accumulated in the brains of mutant mice. Another factor to consider is that we analyzed gene expression in whole brain, not discrete regions. A recent RNA-Seq analysis of basal ganglia from a different model of Slc30a10 deficiency identified greater than 1,000 differentially expressed genes (53). We detected fewer differentially expressed genes, which presumably reflects the fact that we analyzed whole brains.

Another possible explanation for the minimal impact of hepatic Hif2a deficiency on brain gene expression is that hepatic Hif2a deficiency is not sufficient to correct all aspects of Slc30a10 deficiency. This is supported by our recent report in which we injected 1-month-old *Slc30a10^–/–^* mice with adeno-associated virus 8 (AAV8) carrying an *SLC30A10* cDNA under control of the liver-specific thyroxine binding globulin promoter (54). Analysis of mice at 2 months of age revealed attenuation of key phenotypes (tissue Mn excess, liver *Epo* RNA excess) and normalization of several other phenotypes (body mass, serum transaminase levels, liver and whole brain gene expression, RBC parameters). In that study, RNA-Seq–based comparison of livers from *Slc30a10^+/+^* and *Slc30a10^–/–^* mice identified 3,471 differentially expressed genes out of 15,065 total genes detected, while comparison of livers from *Slc30a10^+/+^* and AAV-treated *Slc30a10^–/–^* mice detected only 61 differentially expressed genes out of 12,824 genes assessed. Comparison of whole brains from *Slc30a10^+/+^* and *Slc30a10^–/–^* mice identified 319 differentially expressed genes out of 17,544 total genes detected, while comparison of brains from *Slc30a10^+/+^* and AAV-treated *Slc30a10^–/–^* mice detected no differentially expressed genes out of 17,475 genes assessed. Given that whole brain gene expression in *Slc30a10^–/–^* mice was normalized by hepatic SLC30A10 overexpression but not hepatic Hif2a deficiency, we propose that factors other than Hif2a contribute to the impact of Slc30a10 deficiency on brain phenotypes.

Another point of relevance when considering the use of HIF2 antagonists to treat Mn excess relates to a recent study showing that Mn excess stimulates HIF-dependent *SLC30A10* expression in a homeostatic response to Mn toxicity (20). This study also showed that PHD inhibitors protect mice against Mn toxicity, presumably by upregulating SLC30A10-dependent Mn excretion. We propose that treatment of patients with Mn excess with HIF-modulating agents be considered carefully, as our results suggest that HIF2 inhibition, not stabilization, could be beneficial in SLC30A10 deficiency.

## Methods

### Sex as a biological variable.

Female and male mice were studied.

### Care, generation, and treatment of mice.

Mice were bred and maintained in the animal care facility at Brown University. Mice were group-housed in ventilated cage racks, maintained on a 12-hour light/12-hour dark cycle with controlled temperature and humidity, and provided standard chow (LabDiet 5010 containing 120 ppm Mn) and water ad libitum unless otherwise specified. Littermates of the same sex were randomly assigned to experimental groups. *Slc30a10^–/–^* and *Slc30a10^lox/lox^* mice were generated as previously described (6) and maintained by backcrossing onto C57BL/6N mice (The Jackson Laboratory). *Slc30a10^+/+^* and *Slc30a10^–/–^* mice were generated by crossing *Slc30a10^+/–^* mice. To raise mice on Mn-deficient diets, mice were weaned onto diets containing 1 or 100 ppm Mn (Envigo TD.140497, TD.140499). *Slc39a14^–/–^* mice were generated as previously described (55) and maintained by backcrossing onto 129S6/SvEvTac mice (Taconic Biosciences). To generate mice with Slc30a10 and Slc39a14 deficiency, *Slc30a10^+/–^* and *Slc39a14^+/–^* mice were crossed, and then *Slc30a10^+/–^ Slc39a14^+/–^* progeny were bred together. To generate *Slc30a10^–/–^* mice with hepatocyte Hif1a or Hif2a deficiency, *Slc30a10^+/–^* mice were bred to mice expressing an albumin promotor-driven Cre recombinase transgene (B6N.Cg.*Speer6-ps1^Tg(Alb-Cre)21Mgn^*/J; “*Alb”*; The Jackson Laboratory 018961) and B6.129-*Hif1a^tm3Rsjo^*/J (“*Hif1a^fl/fl^*”; The Jackson Laboratory, 007561) or *Epas1^tm1Mcs^/J* (“*Hif2a^fl/fl^*”; The Jackson Laboratory, 008407) mice in a multistep breeding scheme. Mice for analysis were ultimately generated by crossing *Slc30a10^+/–^ Hif1a^fl/fl^* and *Slc30a10^+/–^ Hif1a^+/fl^ Alb* mice and *Slc30a10^+/–^ Hif2a^fl/fl^* and *Slc30a10^+/–^ Hif2a^+/fl^ Alb* mice. For ASO-based studies, *Slc30a10^+/+^* mice were treated with sterile-filtered PBS, and *Slc30a10^–/–^* mice were treated with sterile-filtered GalNAc control, *Hif1a* ASO, or *Hif2a* ASO (10 μL/g body weight, stock 0.25 mg/mL, Ionis Pharmaceuticals). ASOs were administered by intraperitoneal injection twice a week beginning at weaning until 6 weeks of age for a total of 12 doses. Animals were harvested 2 days after the last dose.

### Bile, blood, and tissue collection.

For mice from which bile was not collected (Figures 1–3; Supplemental Figures 1 and 2; Supplemental Figure 7, A, B, and G–I; and Supplemental Figure 11, A–E), mice were anesthetized by intraperitoneal injection of ketamine and xylazine. Blood was then collected by retro-orbital puncture into EDTA-coated tubes (BD) using heparinized capillary tubes (Thermo Fisher Scientific), then into serum collection tubes (BD) using nonheparinized capillary tubes (Thermo Fisher Scientific). Mice were euthanized by cervical dislocation and tissues collected for metal, DNA, RNA, and protein analysis. (Gastrointestinal tracts were washed of luminal contents.)

For all other mice, mice were anesthetized with isoflurane and then underwent bile, blood, and tissue collection. Bile was collected surgically by ligation of the common bile duct, cannulation of the gallbladder, and collection over 60 minutes as previously described (6). Bile volumes were measured every 5 minutes to permit calculation of bile flow rates. Blood was then collected by cardiac puncture, and mice were transcardially perfused with PBS to remove blood from tissues. Tissues were then collected and processed as above.

### Blood analysis.

Complete blood counts were performed on freshly isolated anticoagulated blood using VetAbcPlus (Sci). Serum Epo and hepcidin levels were measured using Mouse Erythropoietin/EPO Quantikine ELISA Kit (R&D Systems, Bio-Techne) and Hepcidin Murine Complete ELISA Kit (Intrinsic Life Sciences), respectively.

### RNA analysis.

For PCR array analysis, total RNA was extracted using Aurum Total RNA Mini Kit (Bio-Rad). Concentration and purity of extracted RNA were determined by NanoDrop ND-1000 (Thermo Fisher Scientific). Ribosomal RNA integrity was assessed by Agilent Bioanalyzer. RNA (1 μg) was reverse-transcribed to cDNA using iScript Advanced cDNA Synthesis Kit (Bio-Rad). Each 96-well hypoxia array (Bio-Rad) contained primers for 91 hypoxia signaling pathway–related genes and 5 endogenous controls for amplification, DNA contamination, reverse transcription, and RNA quality. qPCR was carried out on Viia 7 Real-Time PCR System (Applied Biosystems, Thermo Fisher Scientific) using SsoAdvanced Universal SYBR Green Supermix (Bio-Rad). Data were exported to Bio-Rad CFX Manager Software and analyzed using relative quantification (2^ΔΔCt^) approach. A 2-fold change in gene expression was used as a cutoff for up- or downregulated genes.

For gene-specific qPCR, 100 to 200 mg tissue was homogenized in TRIzol (Thermo Fisher Scientific) using 0.5 mm zirconium beads and Bullet Blender (Next Advance), followed by chloroform extraction, isopropanol precipitation, and 70% ethanol wash, as previously described (6). Standards were made by serially diluting mixtures of control and experimental samples, then processed identically as experimental samples. Samples underwent DNase treatment and cDNA synthesis using the High Capacity cDNA Reverse Transcription Kit with RNase Inhibitor (Thermo Fisher Scientific). qPCR was performed using PowerUP SYBR Green Master Mix (Thermo Fisher Scientific). Primers listed below were designed using Primer3 and Geneious. Amplicon fidelity was confirmed by Sanger sequencing. Primer concentrations were optimized by testing a series of combination of forward and reverse primers followed by melt curve analyses. The following primers were used: *Hprt1* (5′GCCCCAAAATGGTTAAGGTT3′, 5′TGGCAACATCAACAGGACTC3′, exons 6–9); *Slc30a10* (5′AGAGACTGCTGTCATCTTGCTG3′, 5′TGTGCACTTCATGCACACTG3′, exons 3–4); *Slc39a14* (5′GGAACCCTCTACTCCAACGC3′, 5′ATGGTTATGCCCGTGATGGT3′, exons 5–7); *Anxa2* (5′ATGTCTACTGTCCACGAAATCCT3′, 5′CGAAGTTGGTGTAGGGTTTGACT3′, exons 2–3); *Hk2* (5′TCAAAGAGAACAAGGGCGAG3′, 5′AGGAAGCGGACATCACAATC3′, exons 9–10); *Serpine1* (5′CCTCTTCCACAAGTCTGATGGC3′, 5′GCAGTTCCACAACGTCATACTCG3′, exons 4–5); *Hamp* (5′TGTCTCCTGCTTCTCCTCCT3′, 5′CTCTGTAGTCTGTCTCATCTGTTG3′, exons 1–2); *Epo* (5′ACTCTCCTTGCTACTGATTCCT3′, 5′ATCGTGACATTTTCTGCCTCC3′, exons 2–3); *Hif1a* (5′TGCTCATCAGTTGCCACTTC3′, 5′CCATCTGTGCCTTCATCTCA3′, exons 2–3); *Hif2a* (5′CTTCCTTCGGACACATAAGC3′, 5′CAAGGCTTTCAGGTACAAGT3′, exons 2–3).

For bulk RNA-Seq, RNA extraction, library preparation, sequencing, and analysis was conducted at Azenta Life Sciences as follows. Total RNA was extracted from freshly frozen tissue samples using QIAGEN RNeasy Plus Universal Mini Kit following manufacturer’s instructions. RNA samples were quantified using Qubit 2.0 Fluorometer (Life Technologies, Thermo Fisher Scientific), and RNA integrity was checked using Agilent Technologies TapeStation 4200. RNA-Seq libraries were prepared using the New England Biolabs NEBNext Ultra II RNA Library Prep for Illumina using manufacturer’s instructions. Briefly, mRNAs were initially enriched with oligo-d(T) beads. Enriched mRNAs were fragmented for 15 minutes at 94°C. First-strand and second-strand cDNA were subsequently synthesized. cDNA fragments were end-repaired and adenylated at 3′ ends, and universal adapters were ligated to cDNA fragments, followed by index addition and library enrichment by PCR with limited cycles. The sequencing libraries were validated on the TapeStation and quantified by using Qubit 2.0 Fluorometer (Invitrogen, Thermo Fisher Scientific) as well as by qPCR (KAPA Biosystems). The sequencing libraries were clustered on a flowcell. After clustering, the flowcell was loaded on the Illumina instrument (4000 or equivalent) according to manufacturer’s instructions. The samples were sequenced using a 2 × 150 bp paired-end configuration. Image analysis and base calling were conducted by the control software. Raw sequence data (.bcl files) generated by the sequencer were converted into FASTQ files and demultiplexed using Illumina’s bcl2fastq 2.17 software. One mismatch was allowed for index sequence identification. After investigating the quality of the raw data, sequence reads were trimmed to remove possible adapter sequences and nucleotides with poor quality. The trimmed reads were mapped to the reference genome available on ENSEMBL using the STAR aligner v.2.5.2b. The STAR aligner is a splice aligner that detects splice junctions and incorporates them to help align entire read sequences. BAM files were generated as a result of this step. Unique gene hit counts were calculated by using featureCounts from the Subread package v.1.5.2. Only unique reads that fell within exon regions were counted. After extraction of gene hit counts, the gene hit counts table was used for downstream differential expression analysis. Using DESeq2, a comparison of gene expression between the groups of samples was performed. The Wald test was used to generate *P* values and log_2_ fold-changes. Genes with adjusted *P* < 0.05 and absolute log_2_ fold-changes > 1 were called as differentially expressed genes for each comparison. A principal component analysis (PCA) was performed using the plotPCA function within the DESeq2 R package. The top 500 genes, selected by highest row variance, were used to generate PCA plots. Venn diagrams were generated using the Bioinformatics & Evolutionary Genomics tool (https://bioinformatics.psb.ugent.be/webtools/Venn/). Gene ontology was performed using ShinyGO (http://bioinformatics.sdstate.edu/go/). Heatmaps on rlog-transformed gene counts were generated using Morpheus at https://software.broadinstitute.org/morpheus/

### Immunoblots.

Nuclear proteins from liver samples were isolated using NE-PER Nuclear and Cytoplasmic Extraction Reagents (Thermo Fisher Scientific) with buffers containing Halt Protease Inhibitor Cocktail (Thermo Fisher Scientific) and 1 mM PMSF. Total protein levels were measured using DC protein assay (Bio-Rad). A total of 15–40 μg of nuclear protein was denatured at 99°C for 10 minutes with Laemmli sample buffer containing 10% (v/v) β-mercaptoethanol, resolved onto 4%–20% Criterion TGX Stain-Free Protein Gels (Bio-Rad), and transferred to Immobilon-P PVDF membrane (MilliporeSigma) under cold conditions by wet-transfer method. Blots were blocked for 6 hours in 5% nonfat dry milk prepared in TBS with 0.1% Tween-20, followed by overnight incubation with primary antibody (HIF-1α antibody: Novus Biologicals, Bio-Techne, NB100-449; 1:2,000, lot A7; HIF-2α/EPAS1 antibody: Novus Biologicals, Bio-Techne, NB100-122; 1:1,000, lot C7). Membranes were washed in TBS with Tween (5 times 5 minutes each), then incubated for 2 hours in HRP-linked secondary antibody [HRP-conjugated Affinipure Goat Anti-Rabbit IgG(H+L), Proteintech SA00001-2, 1:5,000], followed by TBS with Tween washes (5 times 5 minutes each). Antibody binding was detected using ECL Prime Western (Thermo Fisher Scientific) and visualized using ChemiDoc Touch (Bio-Rad). For positive and negative controls, Hep3B cells were transfected with *HIF1A* or *HIF2A* siRNAs using DharmaFECT 4 transfection reagent and 40 nM ON-TARGETplus human *HIF1A* or *EPAS1* siRNA (Horizon Discovery) followed by treatment with 100 μM MnCl_2_ for 24 hours. Whole-cell extracts were prepared using 2% SDS lysis buffer with protease cocktail inhibitor and 1 mM PMSF. Cell homogenates were heated at 99°C for 10 minutes with lysis buffer and stored at –20°C until Western blot analyses as described above.

### Metal measurements.

For measurement of tissue total metal levels, 10–200 mg tissue was digested in 1,000 μL 70% trace-metal-grade nitric acid (Thermo Fisher Scientific) at 65°C for 2 hours, then diluted 25-fold in MilliQ water (MilliporeSigma) and analyzed by ICP-OES (Thermo Fisher Scientific iCAP 7400 DUO) or GFAAS (PerkinElmer AAnalyst 600). (If tissues were isolated from perfused mice, tissues were lyophilized for 48 hours using LABCONCO FreeZone 6 Liter Freeze Dry System prior to acid digestion.) For measurement of blood metal levels, samples were digested with 2 volumes nitric acid at 65°C for 2 hours, diluted 25-fold with water, and then analyzed by GFAAS. For measurement of bile metal levels, samples were digested twice with 1 volume nitric acid at 65°C until dry and twice with 1 volume hydrogen peroxide at 65°C until dry, resuspended in 2% nitric acid, and then analyzed by GFAAS. For ICP-OES, a series of standards were analyzed, and sample values were extrapolated from the generated curve. A quality control standard (IV-28, Inorganic Ventures) was run every 10 samples to assess changes in sensitivity. If tissue size was small or metal levels were too low, GFAAS was used. For GFAAS, standards were measured to create a standard curve. To correct for variations in sensitivity, a quality control standard was analyzed every 10 samples (NIST, SRM 1640a). Irrespective of instrumentation, a correction curve was calculated based on quality control analysis and correction factor applied to each sample to control for changes in instrument sensitivity.

For measurement of tissue nonheme iron levels, 10–200 mg tissue was digested in 1 mL 3N hydrochloric acid (Thermo Fisher Scientific)/10% trichloroacetic acid (MilliporeSigma) at 65°C for 2 days, with 30 minutes of vortexing each day, followed by centrifugation (14,000*g* for 10 minutes at room temperature). Iron levels were measured by mixing 10 μL supernatants with 200 μL chromagen (5 volumes MilliQ water; 5 volumes saturated sodium acetate, Thermo Fisher Scientific; 1 volume chromagen stock, consisting of 0.1% bathophenanthroline sulfonate from MilliporeSigma and 1% thioglycolic acid from MilliporeSigma) in a 96-well plate. Iron standards (Thermo Fisher Scientific) were included. After a 10-minute incubation, absorbances were measured at 535 nm. Mock digests without samples were included for this and all other metal analyses.

### ^59^Fe absorption studies.

Mice were fasted in metabolic cages (Tecniplast) for 4 hours to clear the upper gastrointestinal (GI) tract of most contents prior to gavage. To assess iron absorption, each mouse was gavaged with 1 μCi ^59^FeCl_2_ (PerkinElmer) in 2.21 mM FeCl_3_ in 1 M ascorbic acid, filter-sterilized. After gavage, mice were returned to the metabolic cage and harvested after 1 hour. All mice were then euthanized by cervical dislocation, and GI tract from stomach to rectum as well as gallbladder were removed from each mouse. Radioactivity levels were measured using a Triathler Gamma Counter with external NaI well-type crystal detector (Hidex). All samples were counted for 15 seconds. Whole GI tract with contents was counted first in a 15 mL conical tube with stomach positioned closest to the detector. Gallbladder was counted in a 1.5 mL centrifuge tube. Body levels were measured with mice positioned headfirst in a 50 mL conical. After measuring levels in whole GI tract with contents, the whole GI tract was separated into stomach, small intestine, cecum, and large intestine. Each compartment was cleaned out and rinsed. Radioactivity for each rinsed compartment was measured by placing tissue in 1.5 mL centrifuge tube. Percentage absorption was calculated as sum of radioactivity in whole carcass/total radioactivity.

### Statistics.

For all non–RNA-Seq analyses, statistics were performed using GraphPad Prism 9. Data were tested for normal distribution by Shapiro-Wilk test. If not normally distributed, data were log-transformed. Groups within each sex were compared by 1- or 2-way ANOVA with Tukey’s multiple comparisons test or by unpaired, 2-tailed *t* test as indicated in figure legends. *P* < 0.05 was considered significant. Data are represented as means ± standard deviation.

### Study approval.

Mouse studies were approved by the Institutional Animal Care and Use Committee at Brown University.

### Data availability.

All nonsequencing data are contained within the Supporting Data Values XLS file. Regarding sequencing data, BAM files can be found under accessions PRJNA924303 (liver data) and PRJNA1093082 (brain data) on the National Center for Biotechnology Sequence Read Archive.

## Author contributions

MP, SG, MA, and TBB designed the study; MP, JZZ, LC, GSC, CJM, HLK, BD, JAA, and TBB executed experiments and data analysis; SG and MA provided reagents; TBB wrote the manuscript; and all authors revised the manuscript.

## Supplementary Material

Supplemental data

Unedited blot and gel images

Supporting data values

## Version 1 04/23/2024

In-Press Preview

## Version 2 05/22/2024

Electronic publication
