# Supplementary material for: Hepatic HIF2 is a key determinant of manganese excess and polycythemia in SLC30A10 deficiency
Source: JCI Insight. 2024 Apr 23;9(10):e169738. doi: 10.1172/jci.insight.169738 (PMC11141921; doi:10.1172/jci.insight.169738)
Supplement: Supplemental data [file jciinsight-9-169738-s012.pdf]

Hepatic HIF2 is a key determinant of manganese excess and polycythemia in SLC30A10 deficiency

Milankumar Prajapati[1], Jared Z. Zhang[1], Lauren Chiu[1], Grace S. Chong[1], Courtney J. Mercadante[1#], Heather L. Kowalski[1&], Bradley Delaney[1], Jessica A. Anderson[1], Shuling Guo[2], Mariam Aghajan[2], Thomas B. Bartnikas[1*]

[1]Department of Pathology and Laboratory Medicine, Brown University, Providence, Rhode Island, 02912, USA

[2]Ionis Pharmaceuticals, Inc., Carlsbad, CA, 92010, USA

[#]Currently at Ensoma, Boston, MA, 02210, USA

[&]Currently at Myovant Sciences, Brisbane, CA, 94005

*Correspondence: Thomas B. Bartnikas, 70 Ship St. Box GE5, Providence, RI, 02912; 401-863-3478; thomas_bartnikas@brown.edu

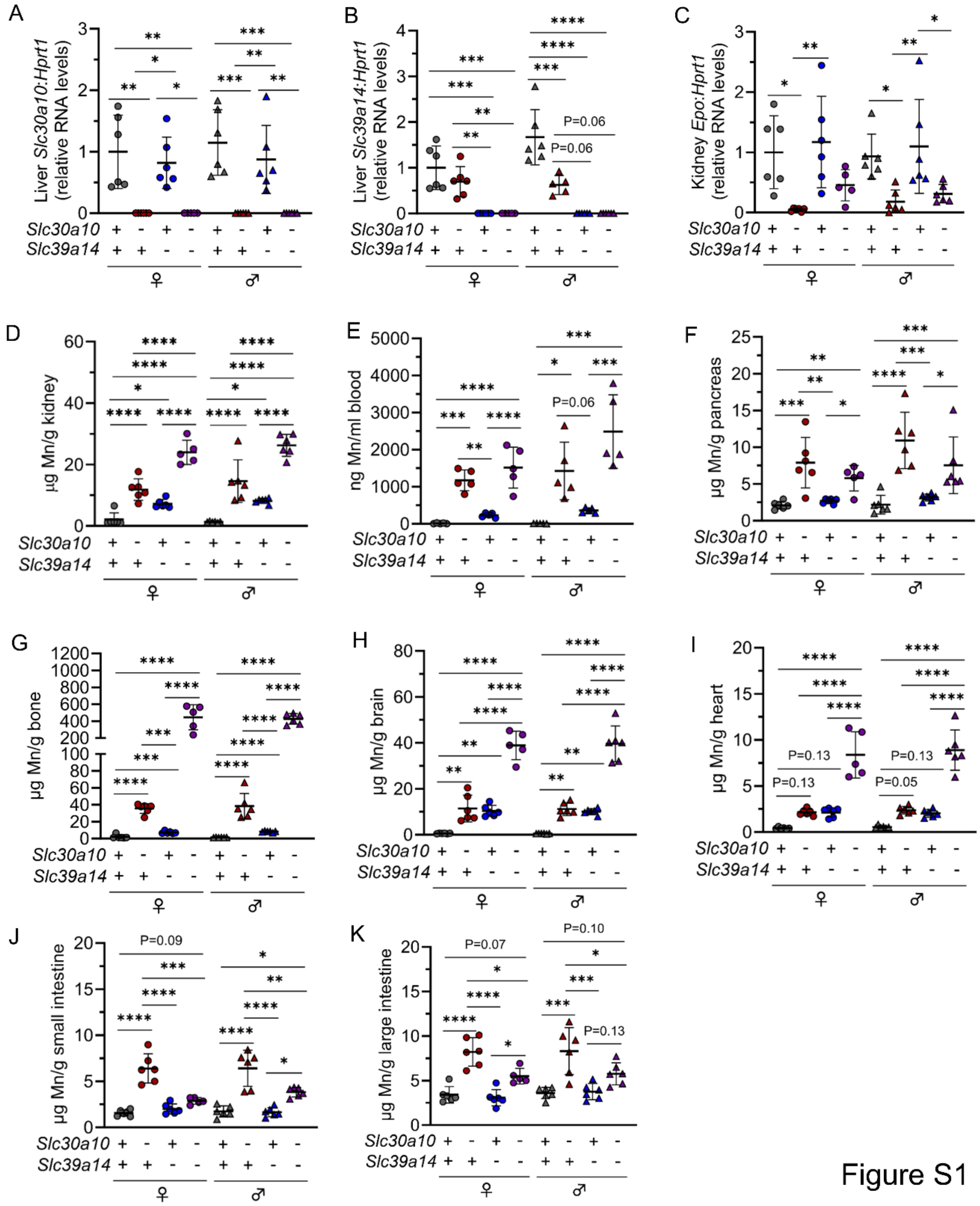

Figure S1

Fig. S1: Slc39a14 deficiency impacts Mn levels in *Slc30a10$^{-/-}$* mice. Five-week-old *Slc30a10 Slc39a14* mice were analyzed for: liver *Slc30a10* (**A**) and *Slc39a14* (**B**) and kidney *Epo* (**C**) RNA levels by qPCR; kidney Mn levels by ICP-OES (**D**); blood Mn levels by GFAAS (**E**); pancreas (**F**), bone (**G**), brain (**H**), heart (**I**), small intestine (**J**), and large intestine (**K**) Mn levels by ICP-OES. Data are represented as means +/- standard deviation, with at least five animals per group. Data were tested for normal distribution by Shapiro-Wilk test; if not normally distributed, data were log transformed. Groups within each sex were compared by two-way ANOVA with Tukey's multiple comparisons test. (ns P=>0.05, * P<0.05, ** P<0.01, *** P<0.001, **** P<0.0001)

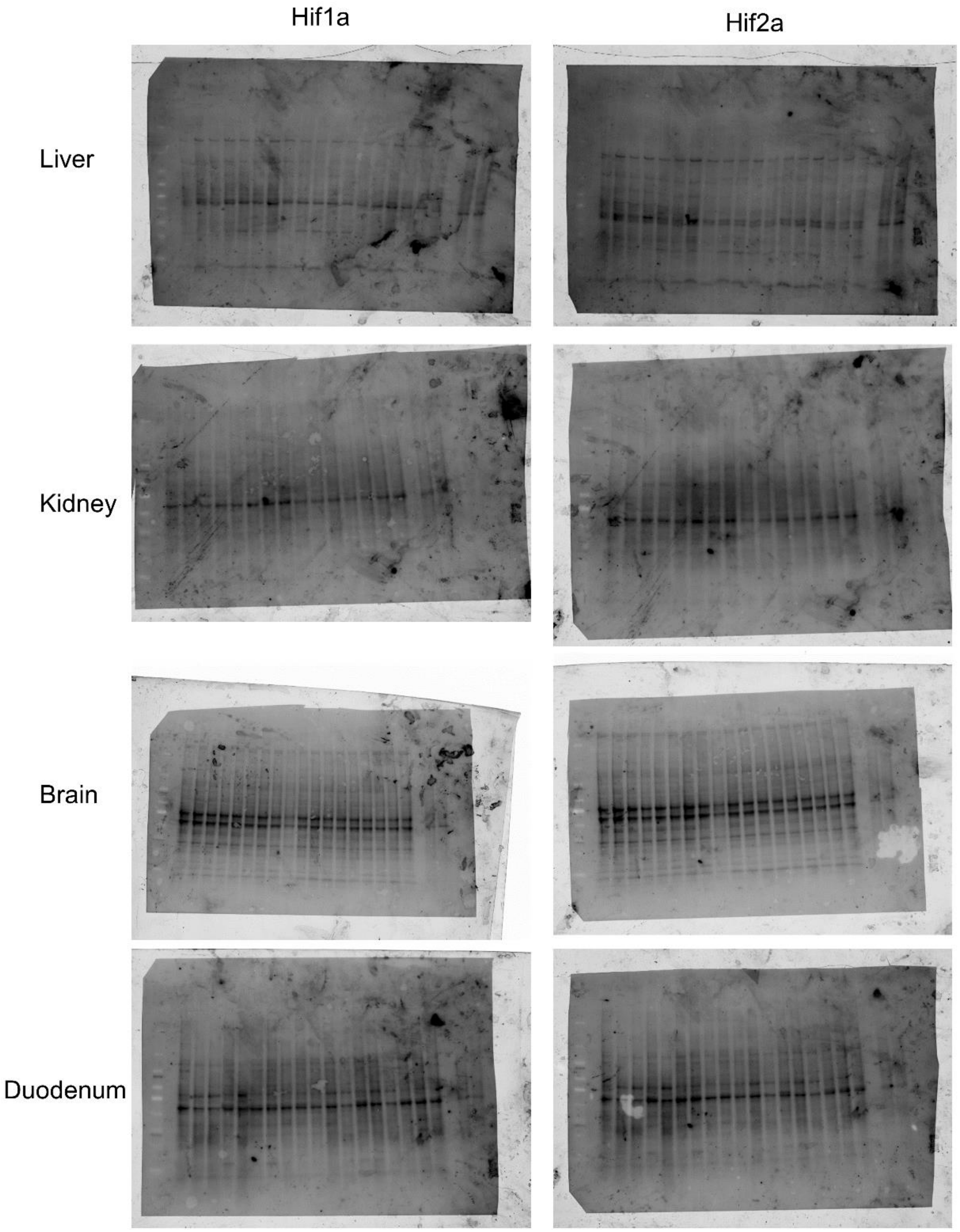

Figure S2

Fig. S2: Hif1a and Hif2a protein levels are increased in livers of *Slc30a10*$^{-/-}$ mice. Two-month-old *Slc30a10* mice were analyzed for Hif1a and Hif2a protein levels by denaturing, reducing immunoblots of nuclear preps from liver, kidney, brain, and duodenum. Total protein images of blots are shown.

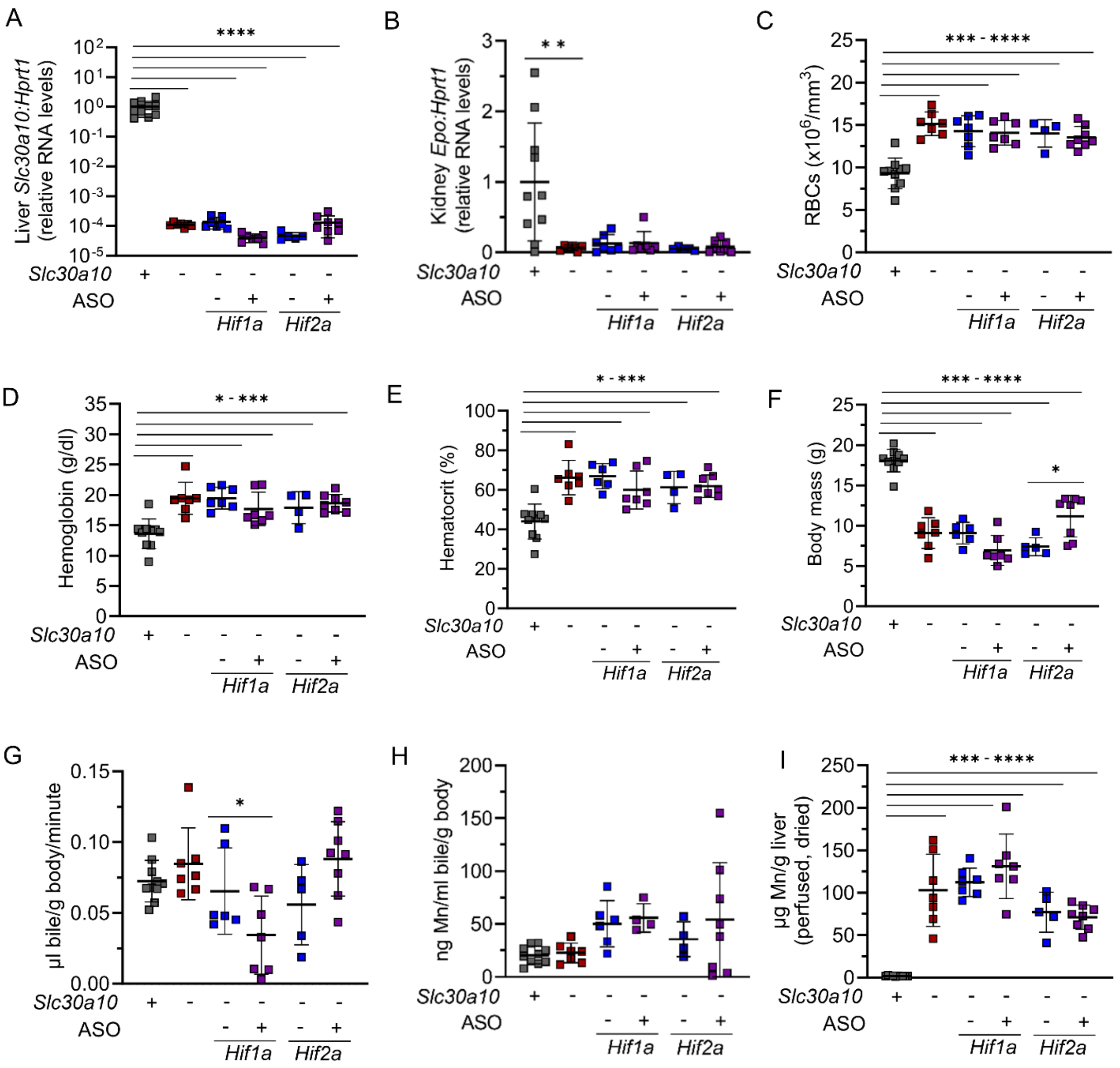

Figure S3

Fig. S3: *Hif2a* ASOs decrease liver *Epo* RNA levels in *Slc30a10*$^{-/-}$ mice. Weanling *Slc30a10*$^{+/+}$ mice were treated with saline and *Slc30a10*$^{-/-}$ mice with GalNAc-conjugated control, *Hif1a*, or *Hif2a* ASOs twice a week for three weeks. Mice were then analyzed for: liver *Slc30a10* (**A**) and kidney *Epo* (**B**) RNA levels by qPCR; RBC counts (**C**), hemoglobin levels (**D**), and hematocrits (**E**) by complete blood counts; body mass (**F**); bile flow rates (**G**); bile Mn levels by GFAAS (**H**); and liver Mn levels by ICP-OES (**I**). Data are represented as means +/- standard deviation, with at least five animals per group. Data were tested for normal distribution by Shapiro-Wilk test; if not normally distributed, data were log transformed. Groups were compared using two-way ANOVA with Tukey's multiple comparisons test. (ns P=>0.05, * P<0.05, ** P<0.01, *** P<0.001, **** P<0.0001)

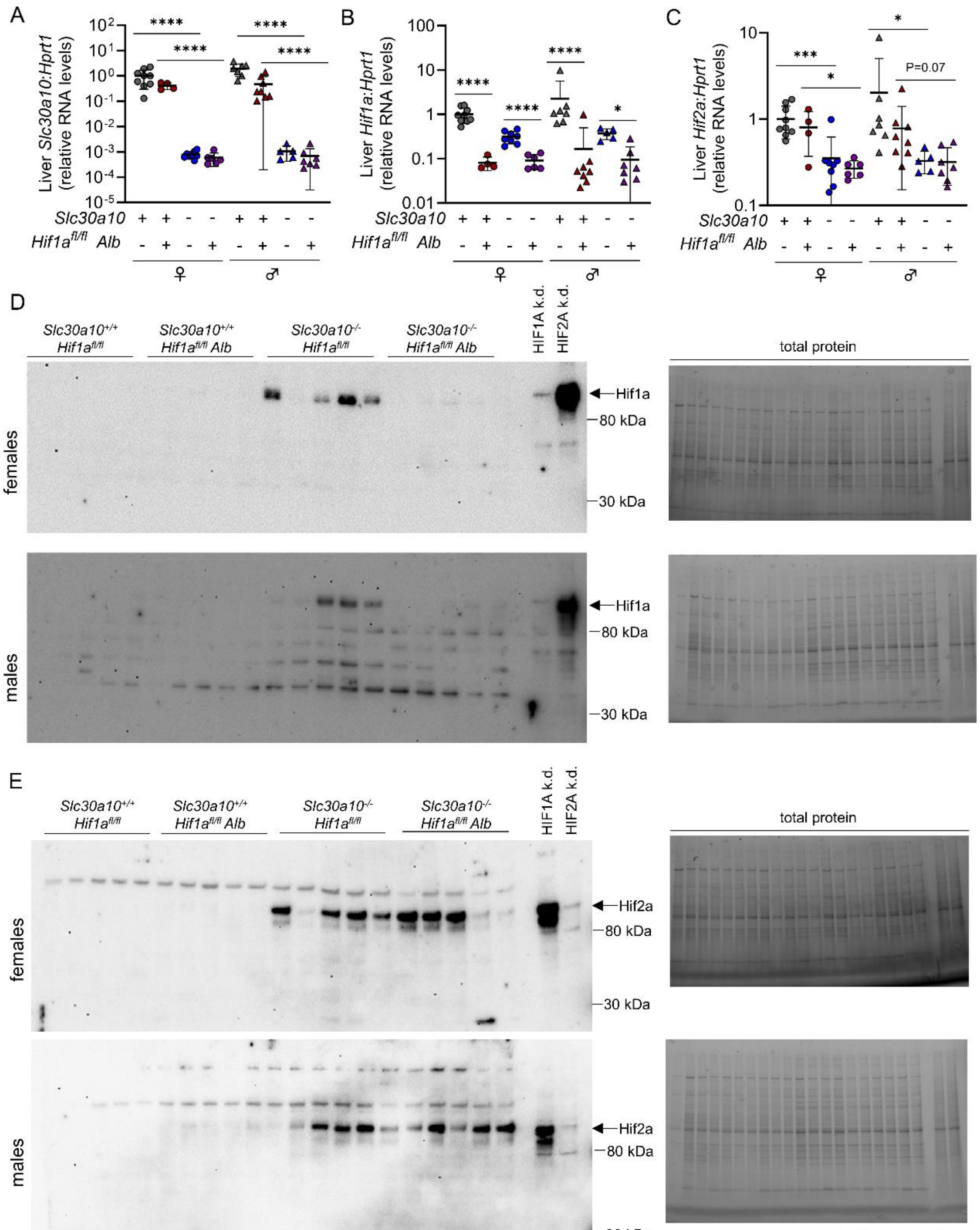

Figure S4

Fig. S4: Hepatocyte Hif1a deficiency decreases Hif1a RNA and protein levels in *Slc30a10*$^{-/-}$ mice. Two-month-old *Slc30a10 Hif1a*$^{fl/fl}$ *+/-Alb* mice were analyzed for: liver *Slc30a10* (**A**), *Hif1a* (**B**), and *Hif2a* (**C**) RNA levels by qPCR; liver Hif1a (**D**) and Hif2a (**E**) protein levels by denaturing, reducing immunoblots of nuclear preps. In (**A-C**), data are represented as means +/- standard deviation, with at least five animals per group. Data were tested for normal distribution by Shapiro-Wilk test; if not normally distributed, data were log transformed. Groups were compared using two-way ANOVA with Tukey's multiple comparisons test. (ns P=>0.05, * P<0.05, ** P<0.01, *** P<0.001, **** P<0.0001) In (**D, E**), two right-most lanes represent nuclear preps from Hep3B cell lines transfected with *HIF1A* ('HIF1A k.d.') or *HIF2A* ('HIF2A k.d.') siRNA then treated with 100 μM $MnCl_2$. Total protein images are shown to the right of blots.

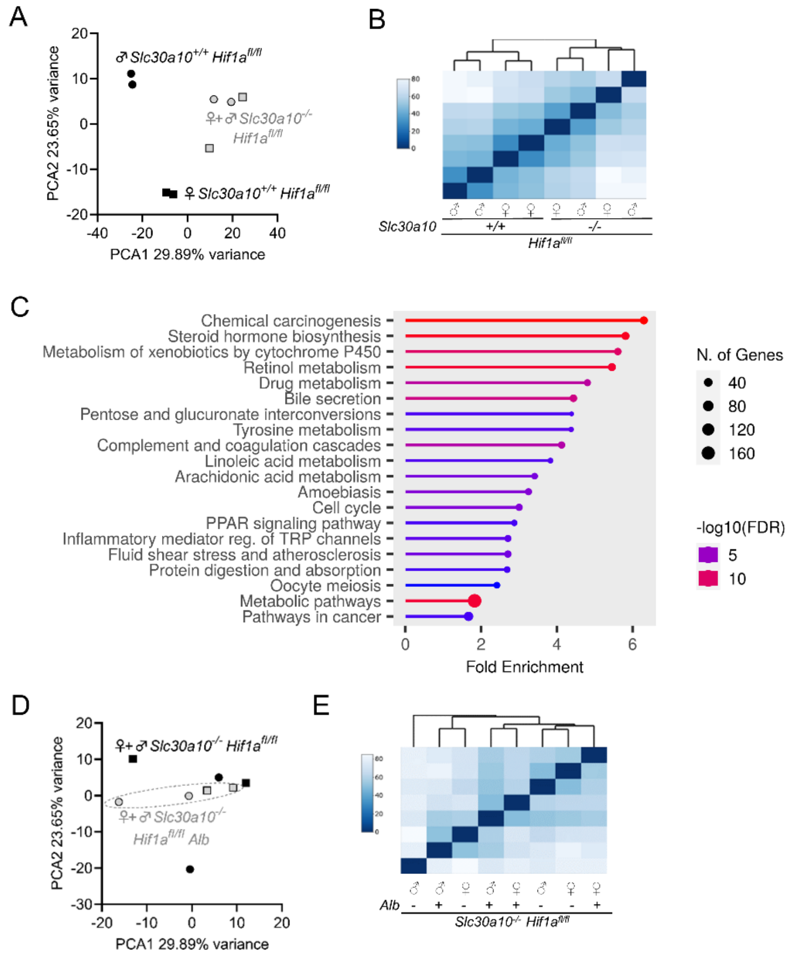

Figure S5

Fig. S5: Hepatic Hif1a deficiency has minimal impact on hepatic gene expression in *Slc30a10$^{-/-}$* mice. (**A-C**) Bulk RNA-seq was performed on livers from two-month-old *Slc30a10$^{-/-}$ Hif1a$^{fl/fl}$* and *Slc30a10$^{+/+}$ Hif1a$^{fl/fl}$* mice. Two females and two males were analyzed per genotype. Shown are principal component (**A**), similarity (**B**), and gene enrichment (**C**) analysis. (**D, E**) Bulk RNA-seq was performed on livers from two-month-old *Slc30a10$^{-/-}$ Hif1a$^{fl/fl}$ Alb* and *Slc30a10$^{-/-}$ Hif1a$^{fl/fl}$* mice. Two females and two males were analyzed per genotype. Shown are principal component (**D**) and similarity (**E**) analysis.

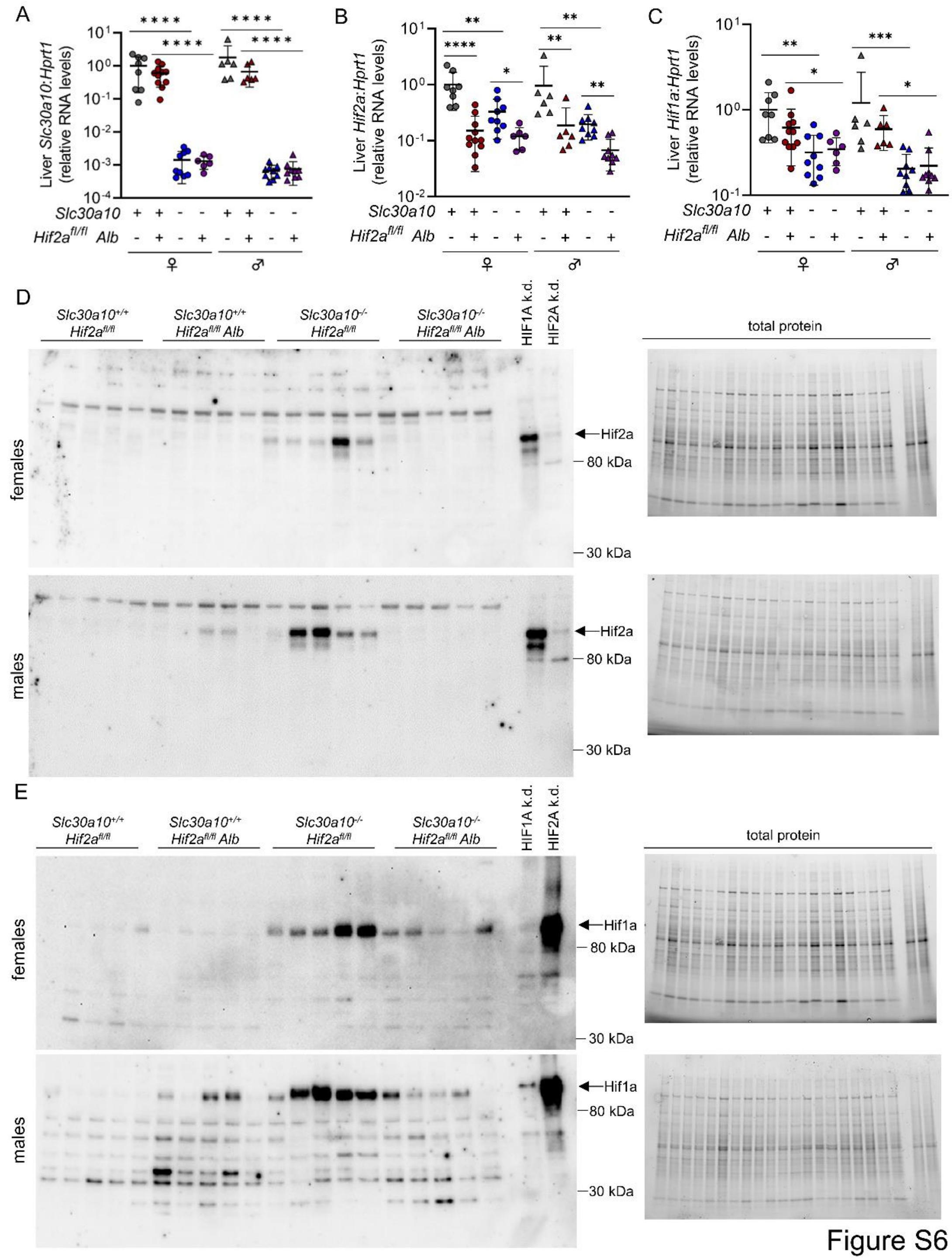

Figure S6

Fig. S6: Hepatocyte Hif2a deficiency decreases Hif2a RNA and protein levels in *Slc30a10$^{-/-}$* mice. Two-month-old *Slc30a10 Hif2a$^{fl/fl}$ +/-Alb* mice were analyzed for: liver *Slc30a10* (**A**), *Hif2a* (**B**), and *Hif1a* (**C**) RNA levels by qPCR; liver Hif2a (**D**) and Hif1a (**E**) protein levels by denaturing, reducing immunoblots of nuclear preps. In (**A-C**), data are represented as means +/- standard deviation, with at least five animals per group. Data were tested for normal distribution by Shapiro-Wilk test; if not normally distributed, data were log transformed. Groups were compared using two-way ANOVA with Tukey's multiple comparisons test. (ns P=>0.05, * P<0.05, ** P<0.01, *** P<0.001, **** P<0.0001) In (**D, E**), two right-most lanes represent 20 µg nuclear preps from Hep3B cell lines transfected with *HIF1A* ('HIF1A k.d.') or *HIF2A* ('HIF2A k.d.') siRNA then treated with 100 µM $MnCl_2$. Total protein images are shown to the right of blots.

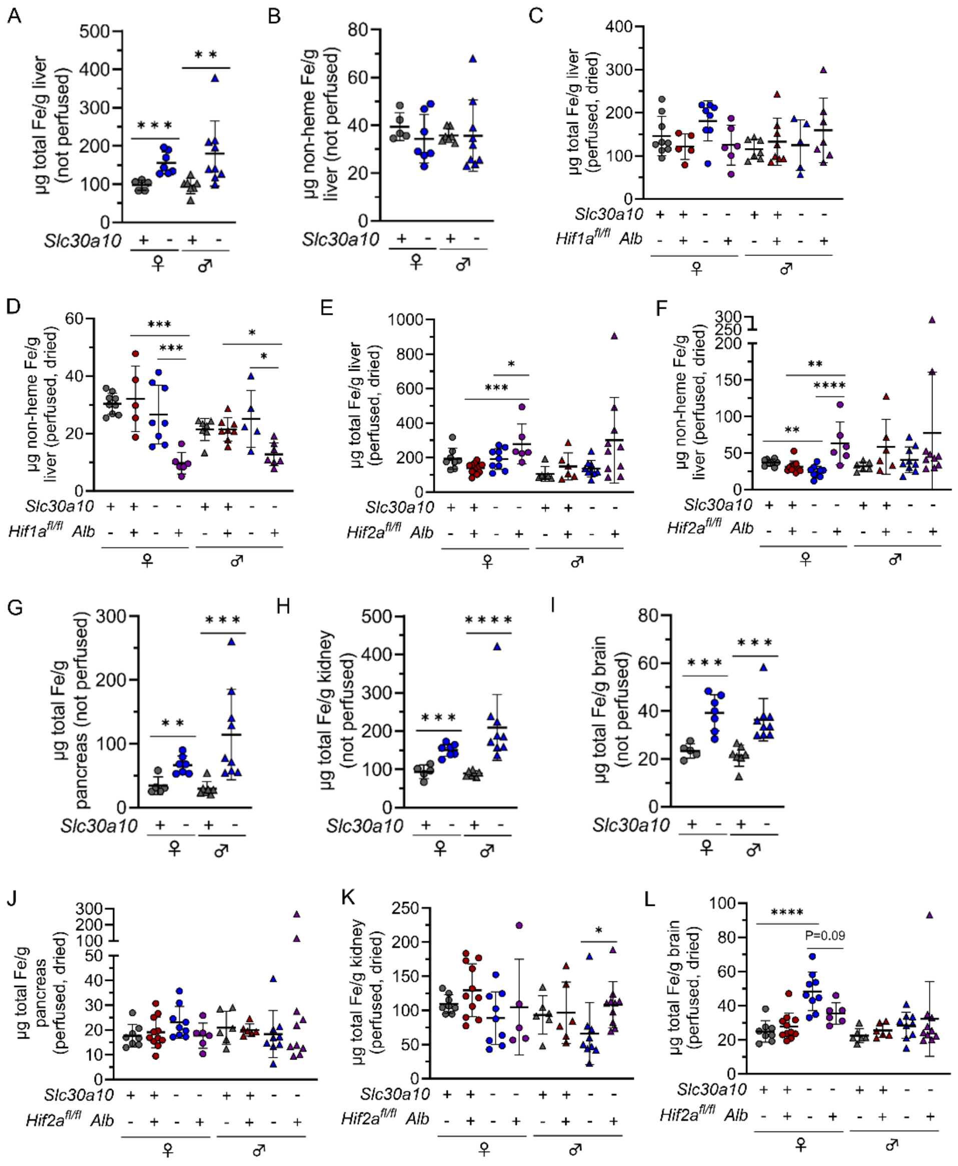

Figure S7

Fig. S7: Perfusion of mice prior to tissue harvest impacts tissue iron (Fe) levels. (**A**) Total Fe levels in livers from two-month-old *Slc30a10*$^{+/+}$ and *Slc30a10*$^{-/-}$ mice, measured by ICP-OES and previously published (6), are reproduced here for reference. (**B**) Non-heme Fe levels were measured by bathophenanthroline-based assay in livers from two-month-old *Slc30a10*$^{+/+}$ and *Slc30a10*$^{-/-}$ mice described in (6). (**C-F**) Total (**C, E**) and non-heme (**D, F**) Fe levels were measured in two-month-old *Slc30a10 Hif1a* (**C, D**) and *Slc30a10 Hif2a* (**E, F**) mice. (**G-I**) Total Fe levels in pancreas (**G**), kidney (**H**), and brain (**I**) from two-month-old *Slc30a10*$^{+/+}$ and *Slc30a10*$^{-/-}$ mice, measured by ICP-OES and previously published (6), are reproduced here for reference. (**J-L**) Total Fe levels in pancreas (**J**), kidney (**K**), and brain (**L**) from *Slc30a10 Hif2a* mice measured by ICP-OES. Note that mice represented in (**A, B, G-I**) were not perfused with saline prior to tissue harvest; for all other panels, mice were perfused with saline and tissues dried prior to metal analysis. In all panels, data are represented as means +/- standard deviation, with at least five animals per group. Data were tested for normal distribution by Shapiro-Wilk test; if not normally distributed, data were log transformed. Within each sex, groups were compared using unpaired, two-tailed tests (**A, B, G, H, I**) or two-way ANOVA with Tukey's multiple comparisons test (**C-F, J-L**). (ns P=>0.05, * P<0.05, ** P<0.01, *** P<0.001, **** P<0.0001)

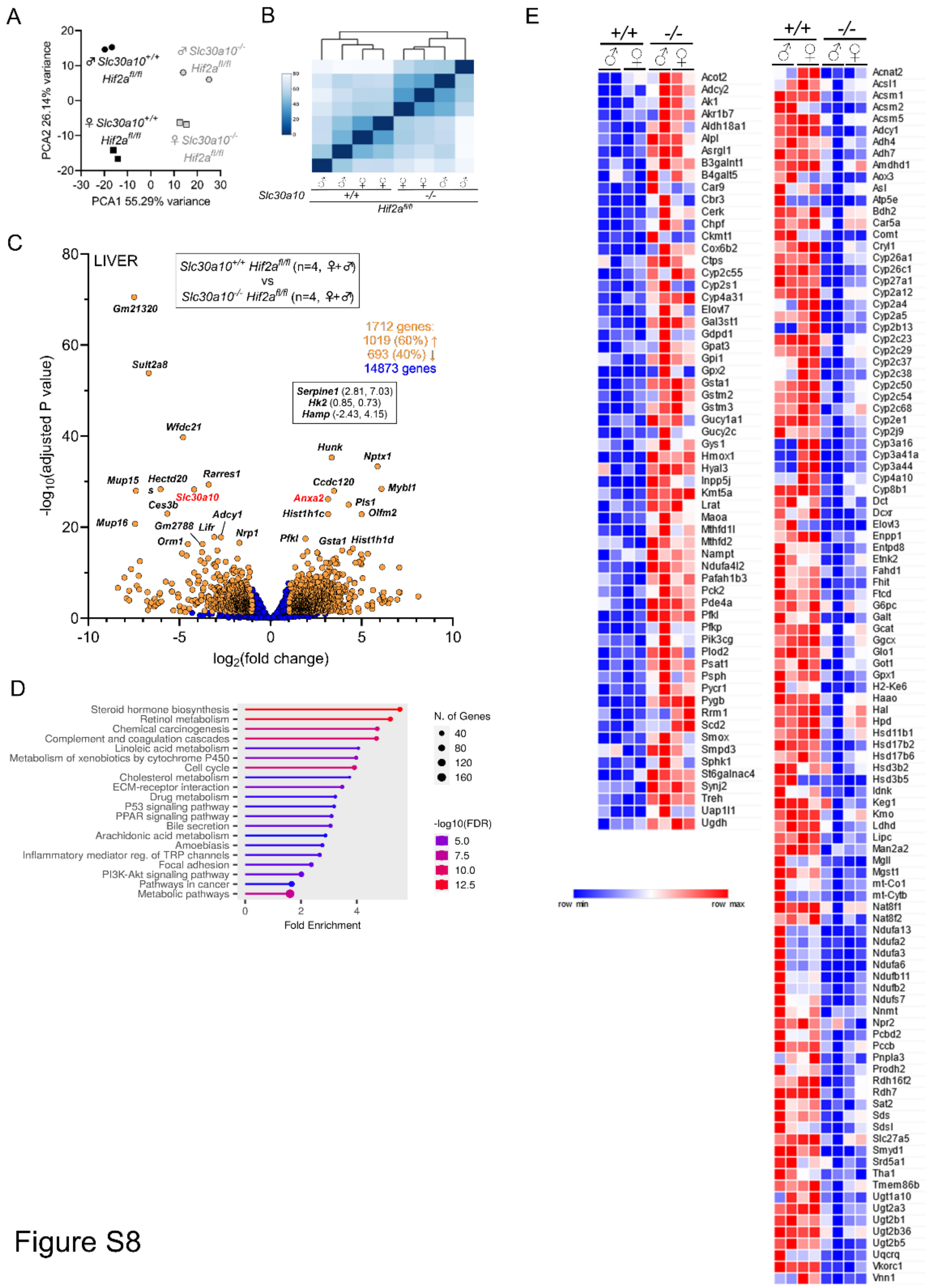

Figure S8

Fig. S8: Slc30a10 deficiency impacts hepatic gene expression. Bulk RNA-seq was performed on livers from *Slc30a10*$^{-/-}$ *Hif2a*$^{fl/fl}$ and *Slc30a10*$^{+/+}$ *Hif2a*$^{fl/fl}$ mice. Shown are: principal component analysis (**A**); similarity analysis (**B**); volcano plot (**C**); gene enrichment analysis (**D**); heatmap of genes aligning with metabolic pathways (**E**). In volcano plot, differentially expressed genes (adjusted P value<0.05 and absolute value of $\log_2$(fold change)>1) are shown as light orange points with gene names shown adjacent as space permitted; non-differentially expressed genes are shown as blue points; x-y coordinates of additional genes of interest are shown in smaller box. Genes with $\log_2$(fold change)<0 are more abundantly expressed in first group listed in box at top of plot; genes with $\log_2$(fold change)>0 are more abundantly expressed in second group listed.

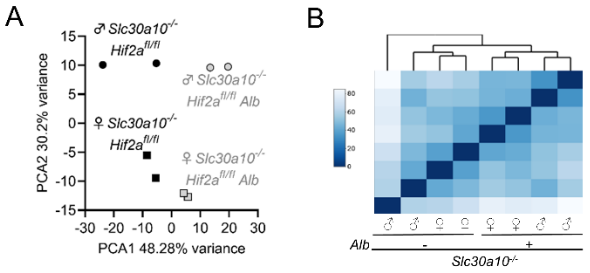

Figure S9

Fig. S9: Hepatocyte Hif2a deficiency attenuates differential gene expression in livers of *Slc30a10*$^{-/-}$ mice. Bulk RNA-seq was performed on livers from two-month-old *Slc30a10*$^{-/-}$ *Hif2a*$^{fl/fl}$ *Alb* and *Slc30a10*$^{-/-}$ *Hif2a*$^{fl/fl}$ mice. Two females and two males were analyzed per genotype. Shown are principal component (**A**) and similarity (**B**) analysis.

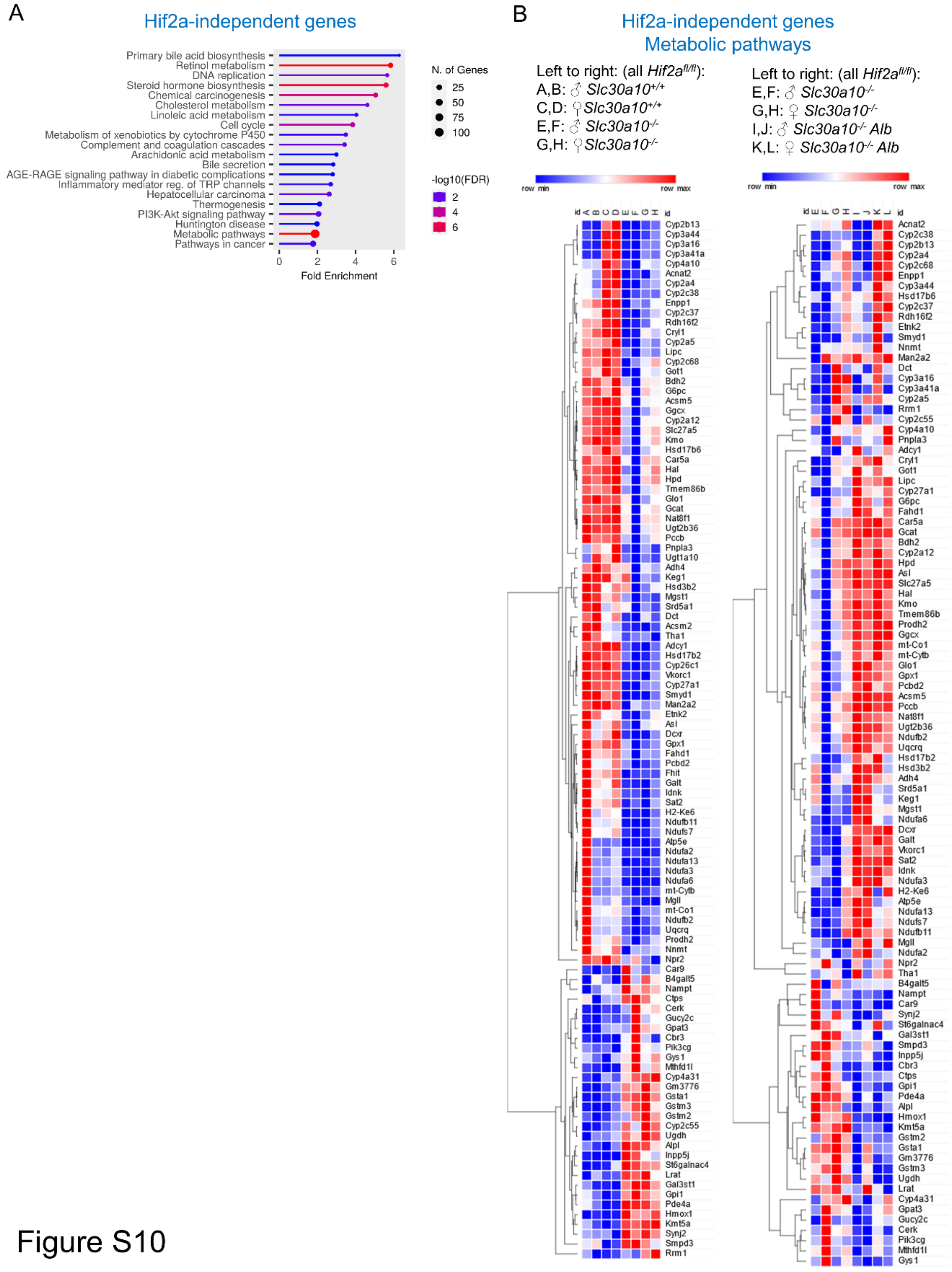

Figure S10

Fig. S10: Differential gene expression in *Slc30a10*$^{-/-}$ livers is Hif2a-independent and -dependent. Bulk RNA-seq was performed on livers from two-month-old *Slc30a10*$^{-/-}$ *Hif2a*$^{fl/fl}$ *Alb* and *Slc30a10*$^{-/-}$ *Hif2a*$^{fl/fl}$ mice. Two females and two males were analyzed per genotype. Shown are (**A**) gene enrichment analysis of Hif2a-independent genes and (**B**) heatmaps of Hif2a-independent genes aligning with metabolic pathways.

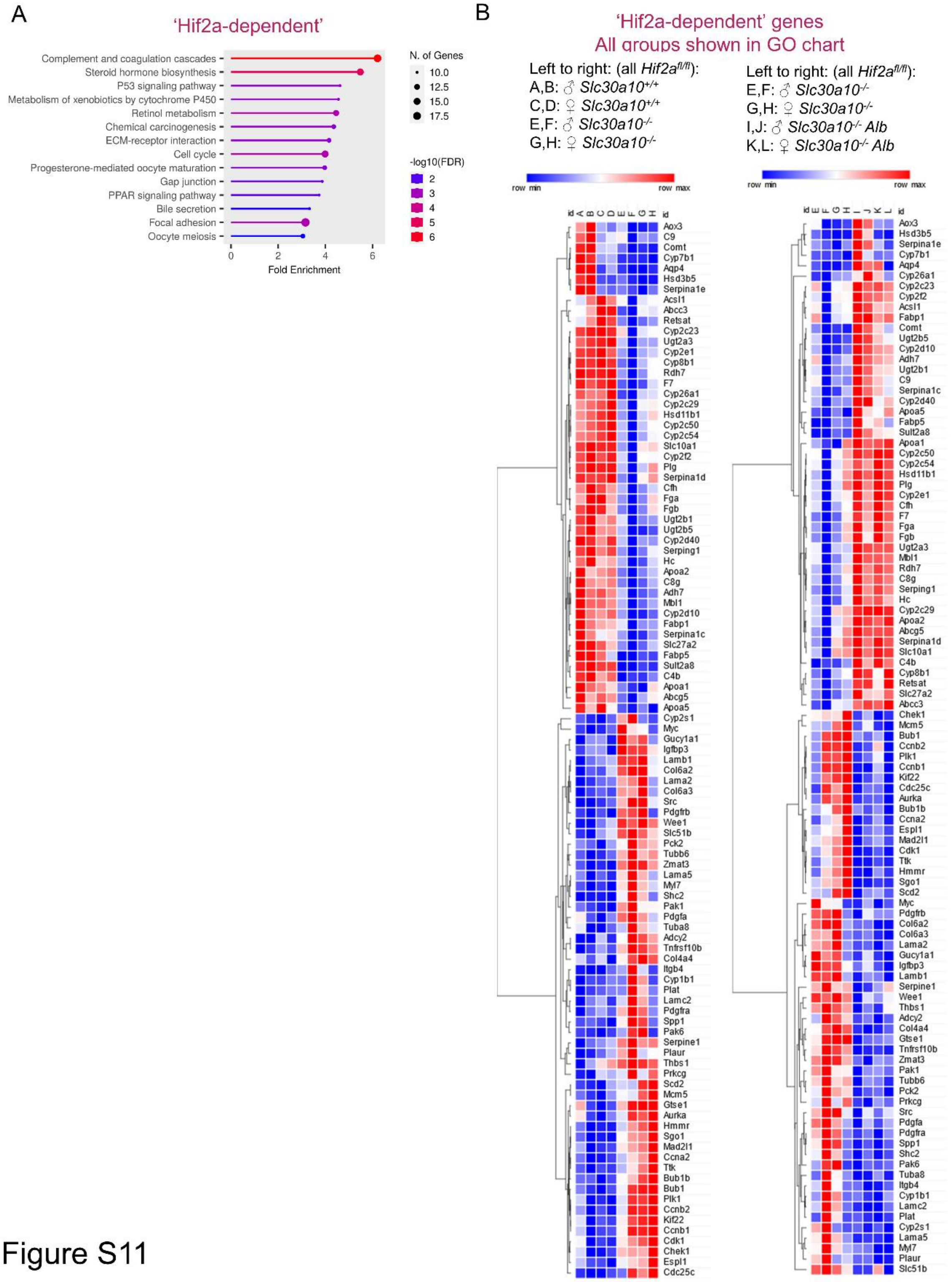

Figure S11

Fig. S11: Differential gene expression in *Slc30a10*$^{-/-}$ livers is Hif2a-independent and -dependent. Bulk RNA-seq was performed on livers from two-month-old *Slc30a10*$^{-/-}$ *Hif2a*$^{fl/fl}$ *Alb* and *Slc30a10*$^{-/-}$ *Hif2a*$^{fl/fl}$ mice. Two females and two males were analyzed per genotype. Shown are gene enrichment analysis (**A**) and heatmaps of Hif2a-dependent genes (**B**).

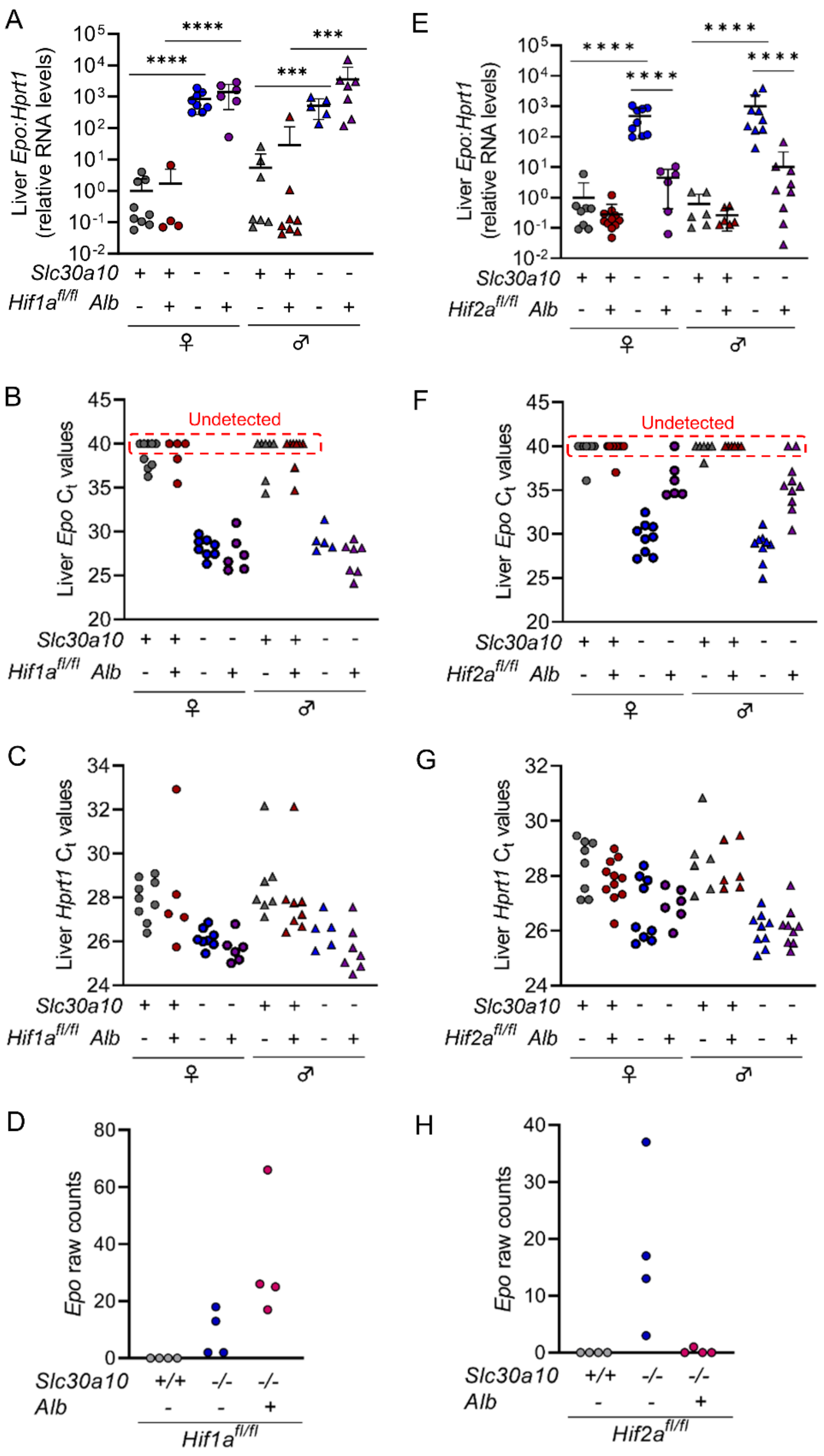

Figure S12

Fig. S12: qPCR C_t values and RNA-seq raw counts for *Epo*. (**A-D**) Reproduction of liver *Epo* RNA levels from Fig. 5A (**A**), along with *Epo* (**B**) and *Hprt1* (**C**) Ct values from qPCR and *Epo* raw counts from RNA-seq (**D**). (**E-H**) Reproduction of liver *Epo* RNA levels from Fig. 7E (**E**), along with *Epo* (**F**) and *Hprt1* (**G**) Ct values from qPCR and *Epo* raw counts from RNA-seq (**H**).

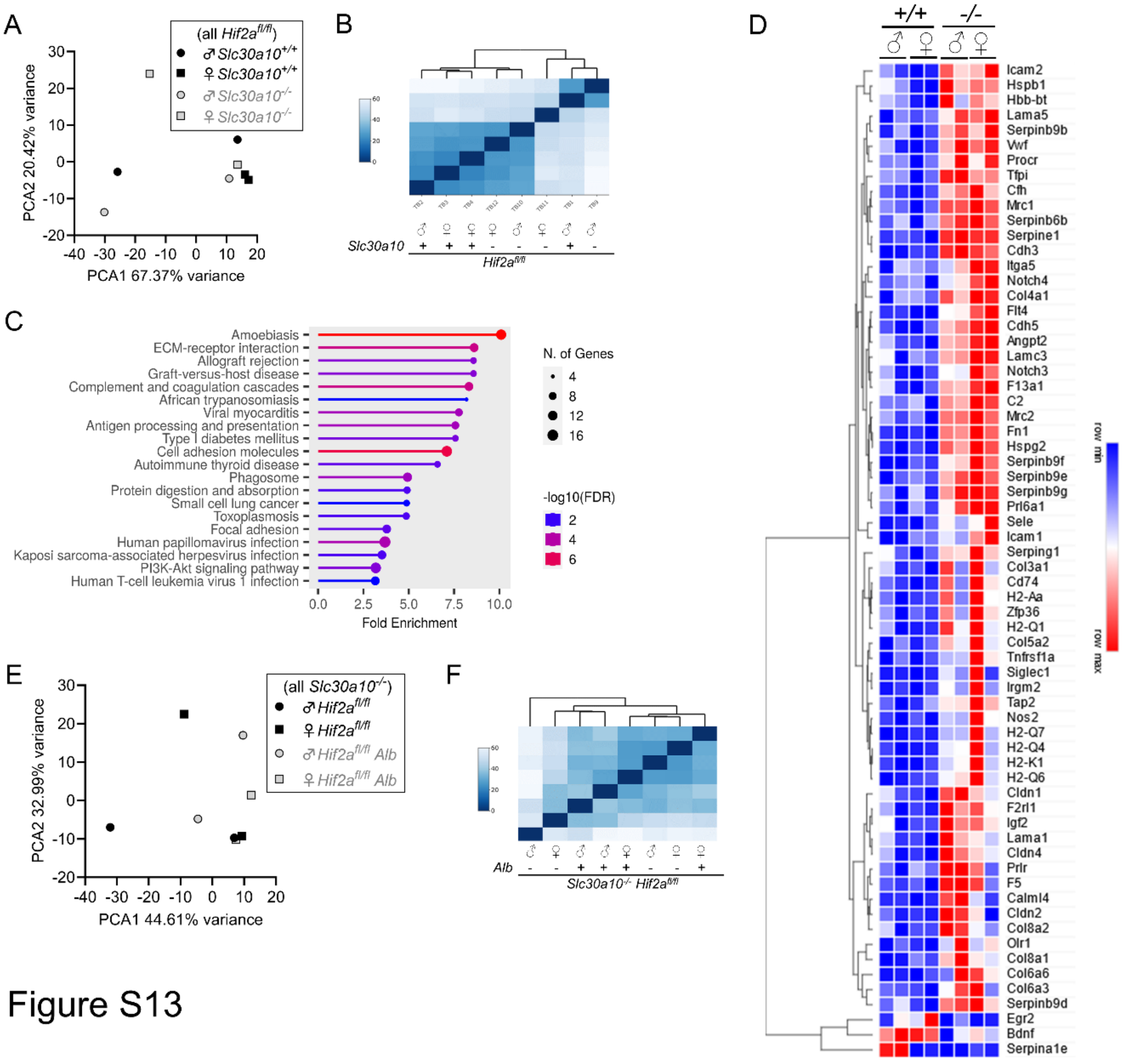

Figure S13

Fig. S13: Hepatic Hif2a deficiency has minimal impact on brain gene expression in *$Slc30a10^{-/-}$* mice. (**A-D**) Bulk RNA-seq was performed on brains from two-month-old *$Slc30a10^{-/-}$ $Hif2a^{fl/fl}$* and *$Slc30a10^{+/+}$ $Hif2a^{fl/fl}$* mice. Two females and two males were analyzed per genotype. Shown are principal component analysis (**A**), similarity analysis (**B**), gene enrichment analysis (**C**), and heatmap of all genes represented in gene enrichment analysis (**D**). (**E, F**) Bulk RNA-seq was performed on brains from two-month-old *$Slc30a10^{-/-}$ $Hif2a^{fl/fl}$ Alb* and *$Slc30a10^{-/-}$ $Hif2a^{fl/fl}$* mice. Two females and two males were analyzed per genotype. Shown are principal component (**E**) and similarity (**F**) analysis.
