# Supplementary material for: Hepatic HIF2 is a key determinant of manganese excess and polycythemia in SLC30A10 deficiency
Source: JCI Insight. 2024 Apr 23;9(10):e169738. doi: 10.1172/jci.insight.169738 (PMC11141921; doi:10.1172/jci.insight.169738)
Supplement: Unedited blot and gel images [file jciinsight-9-169738-s013.pdf]

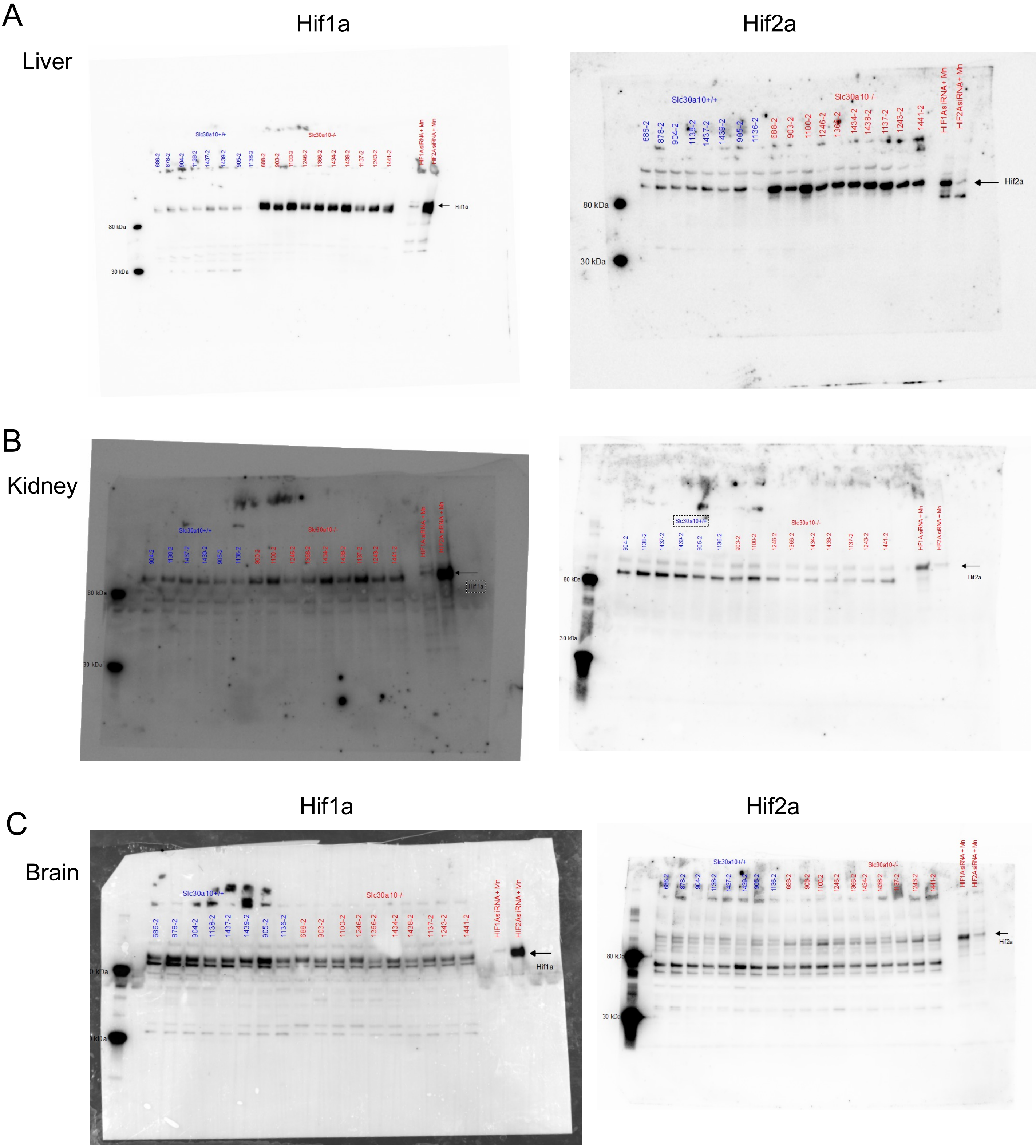

A
Liver
Hif1a
Hif2a
Slc30a10+/+
Slc30a10-/-
686-2
878-2
904-2
1138-2
1437-2
1439-2
905-2
1136-2
688-2
903-2
1100-2
1246-2
1366-2
1434-2
1438-2
1137-2
1243-2
1441-2
HIF1AsiRNA+ Mn
HIF2AsiRNA+ Mn
80 kDa
30 kDa
B
Kidney
C
Brain

Figure 3

D

Small intestine

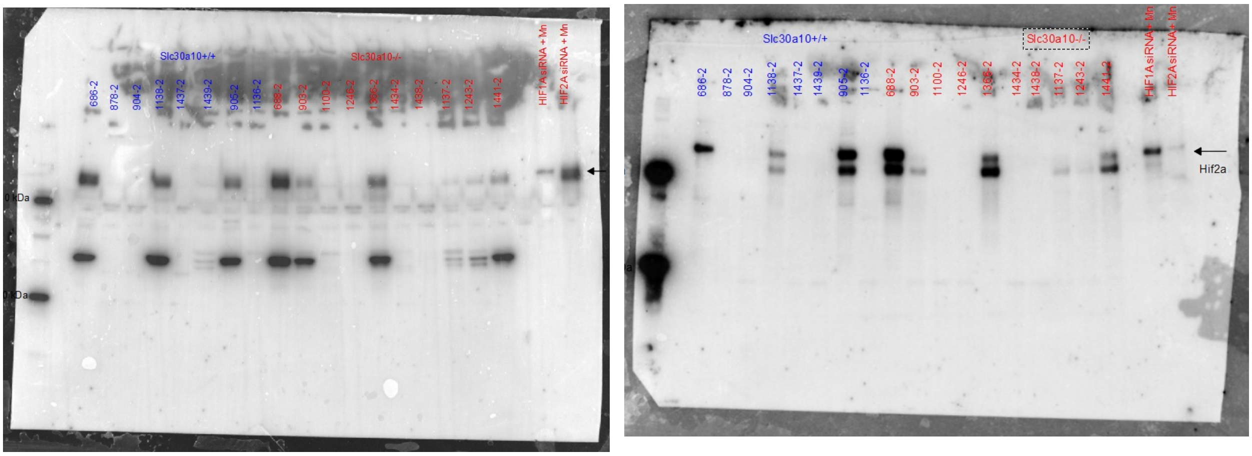

Figure 3

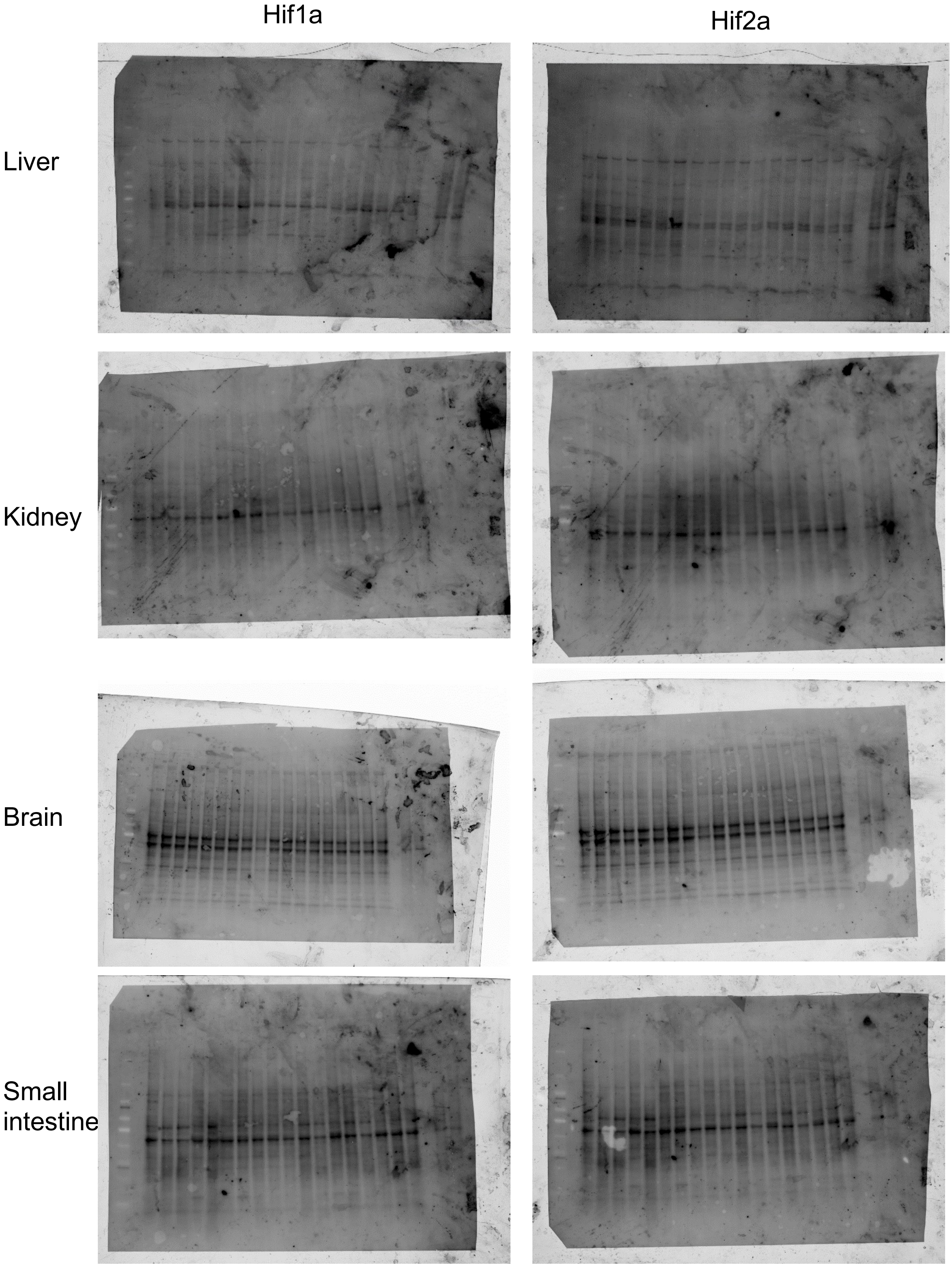

Figure S2

D

Hif1a

females

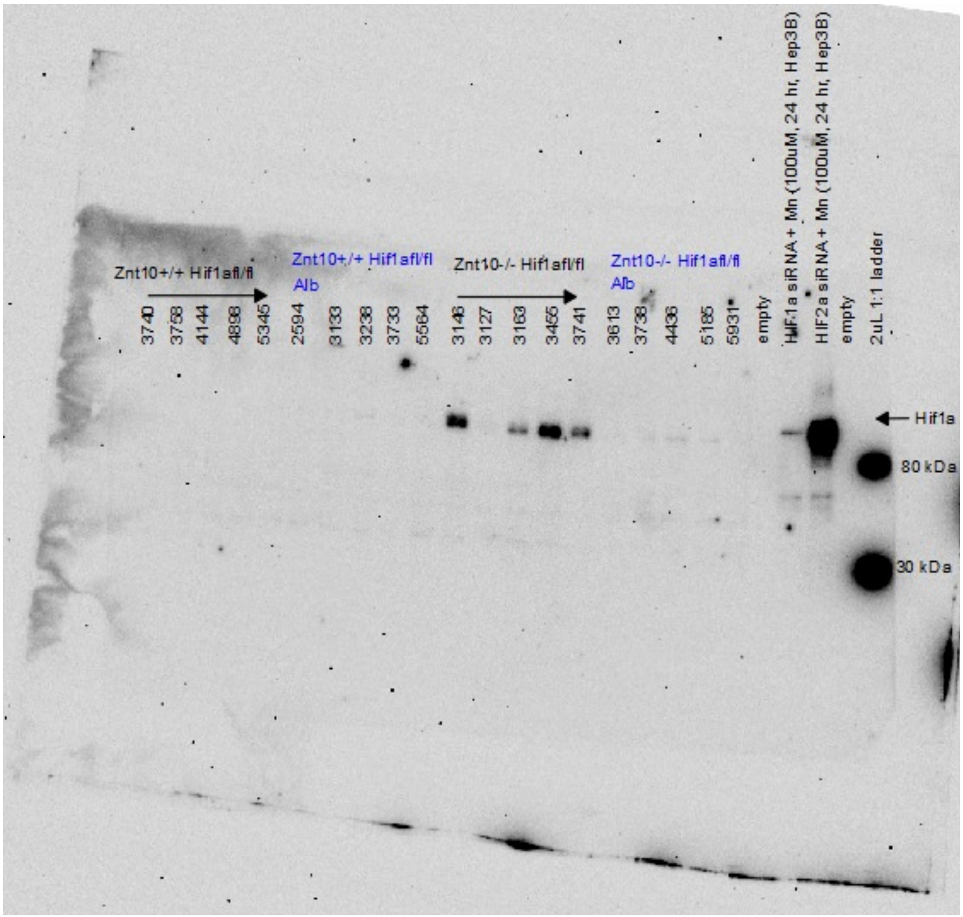

males

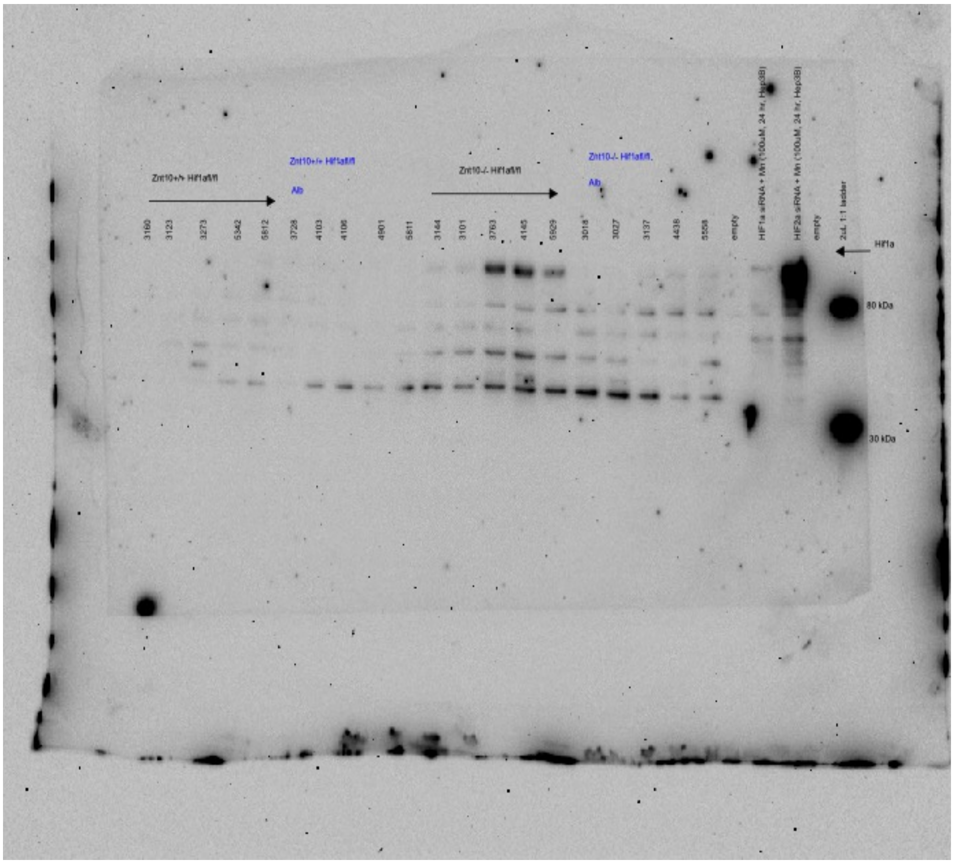

Figure S4

E

Hif2a

females

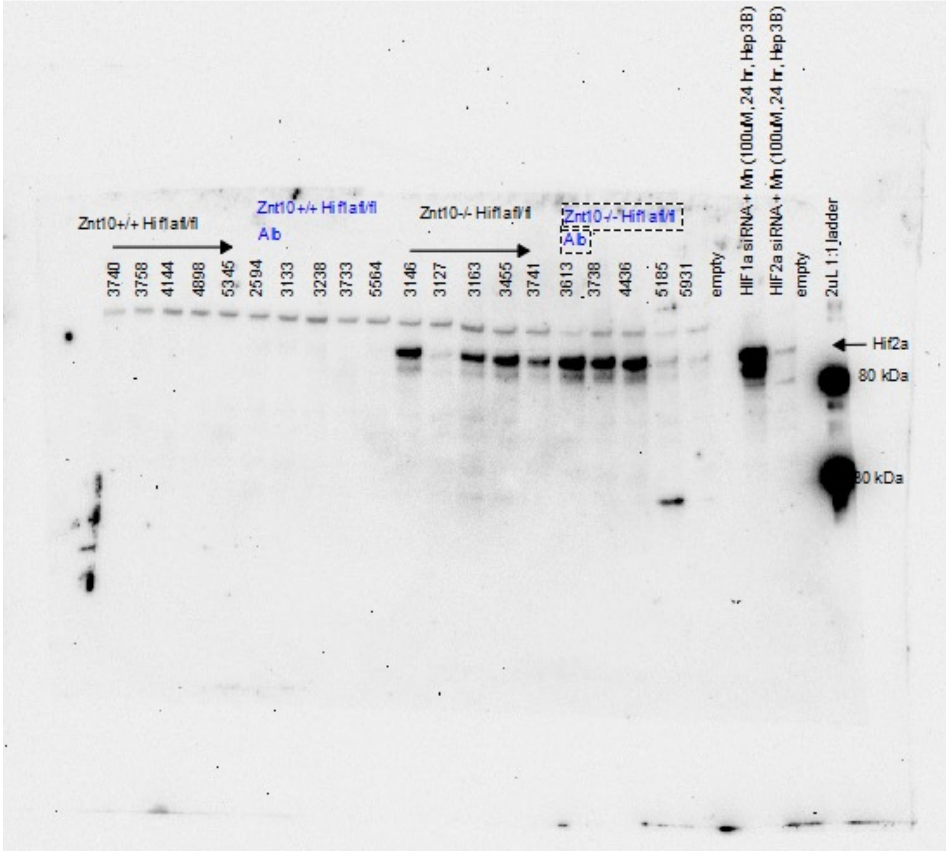

males

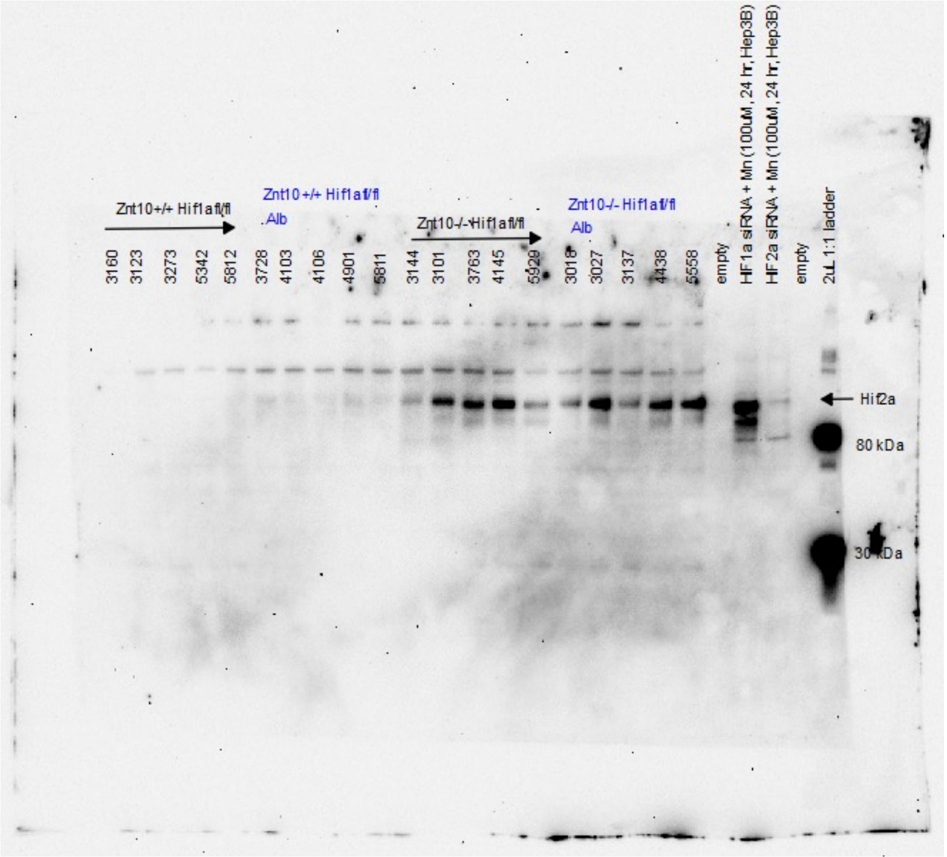

Figure S4

D

Hif2a

females

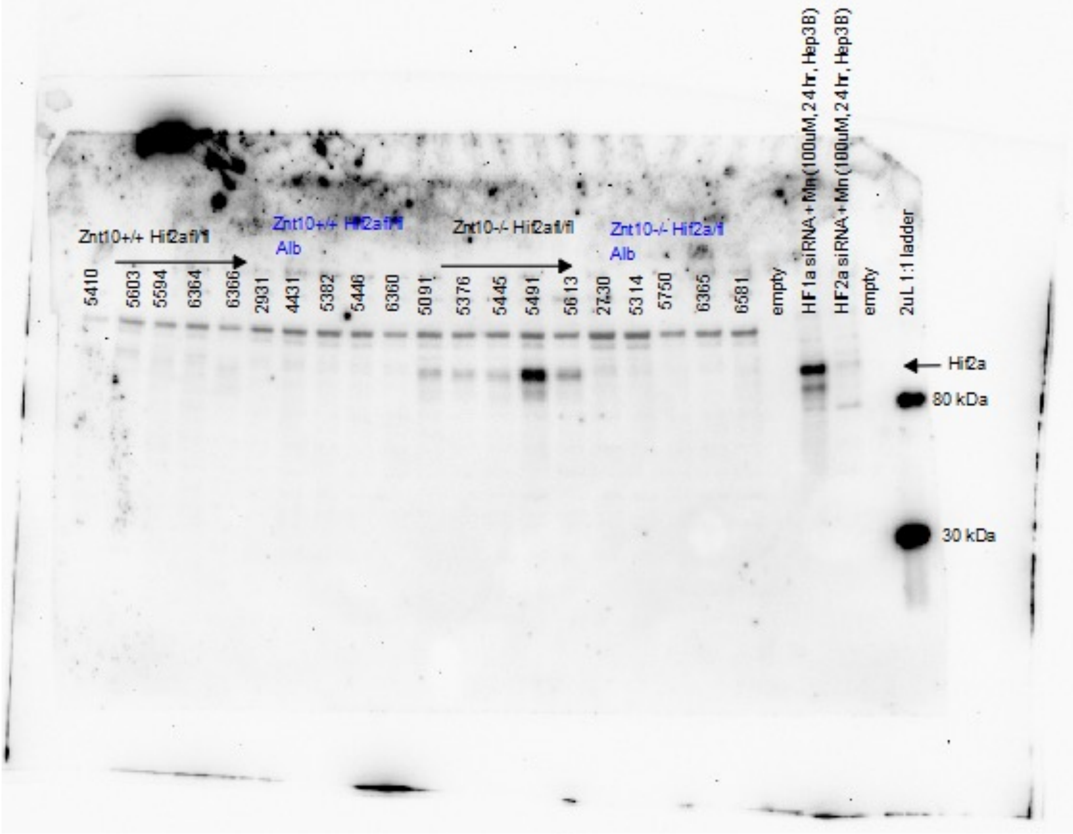

males

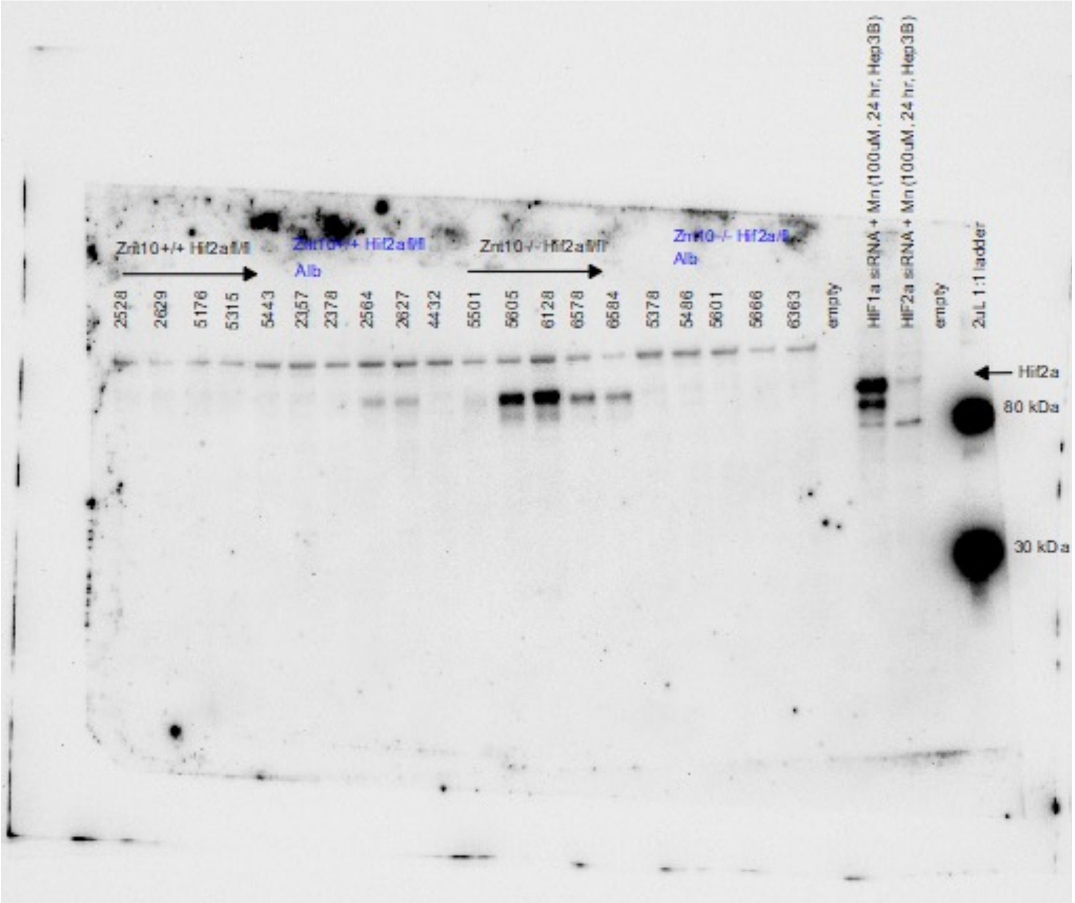

Figure S6

E

Hif1a

females

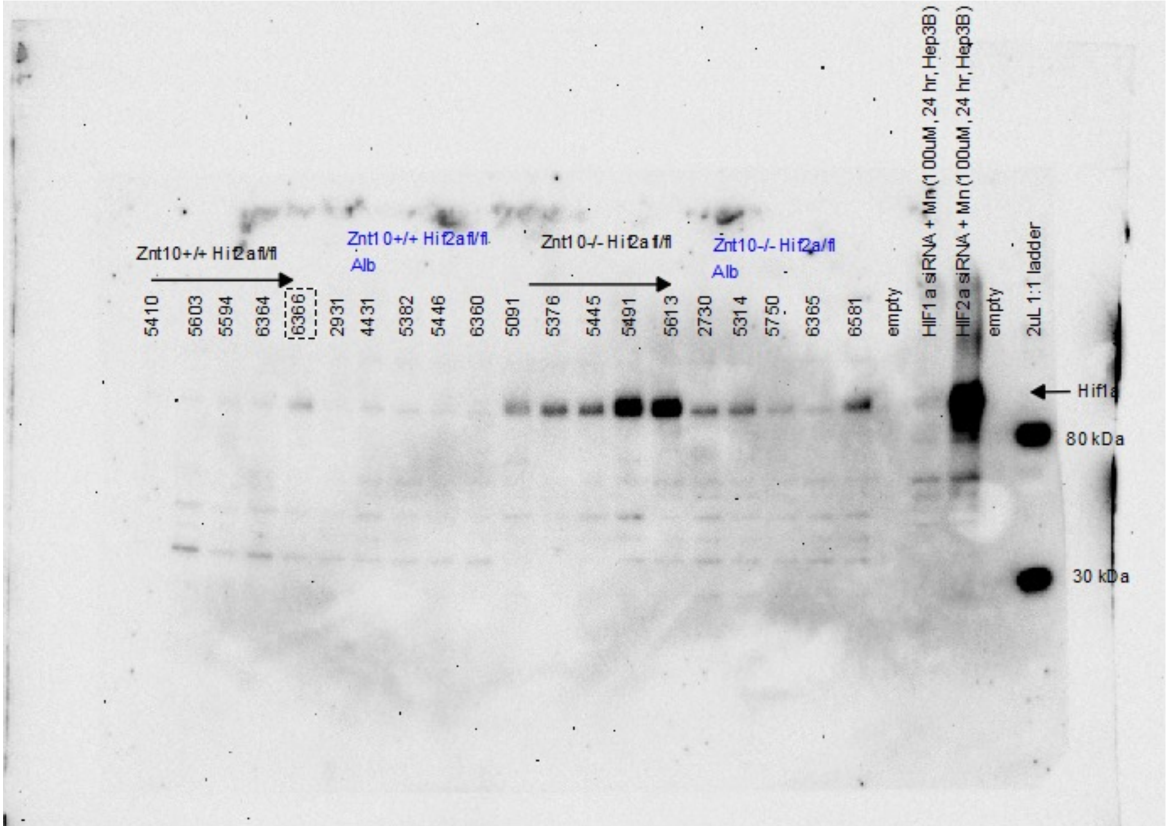

males

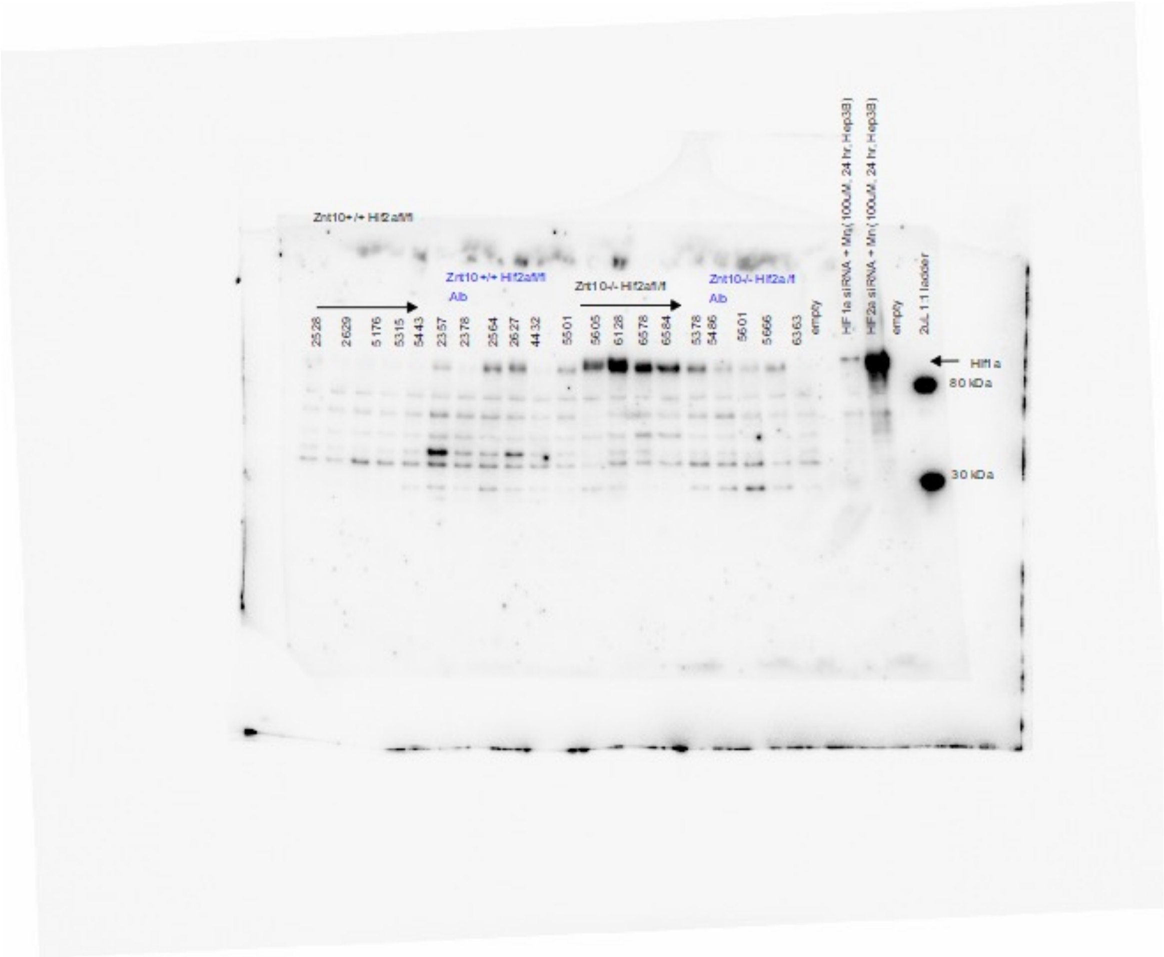

Figure S6
